# The NTPase activity of the double FYVE domain–containing protein 1 regulates lipid droplet metabolism

**DOI:** 10.1016/j.jbc.2022.102830

**Published:** 2022-12-24

**Authors:** V.A. Ismail, T. Naismith, D.J. Kast

**Affiliations:** Department of Cell Biology and Physiology, Washington University School of Medicine, St Louis, Missouri, USA

**Keywords:** ATPase, autophagy, DFCP1, fatty acid, GTPase, lipid droplets, membrane contact site, metabolism, ZFYVE1, BSA, bovine serum albumin, CV, column volume, DFCP1, double FYVE domain–containing protein 1, DMSO, dimethyl sulfoxide, ER, endoplasmic reticulum, ERB, endoplasmic reticulum–binding domain, FA, fatty acid, FBS, fetal bovine serum, FFA, free fatty acid, GIMAP, GTPases of Immunity Associated Protein, LD, lipid droplet, OA, oleic acid, OCR, oxygen consumption rate, PI3P, phosphoinositol-3-phosphate, TAG, triacylglyceride

## Abstract

Lipid droplets (LDs) are transient lipid storage organelles that can be readily tapped to resupply cells with energy or lipid building blocks and therefore play a central role in cellular metabolism. However, the molecular factors and underlying mechanisms that regulate the growth and degradation of LDs are poorly understood. It has emerged that proteins that establish contacts between LDs and the endoplasmic reticulum play a critical role in regulating LD metabolism. Recently, the autophagy-related protein, double FYVE domain–containing protein 1 (DFCP1/ZFYVE1) was shown to reside at the interface of the endoplasmic reticulum and LDs, however, little is known about the involvement of DFCP1 in autophagy and LD metabolism. Here, we show that DFCP1 is a novel NTPase that regulates free fatty acid metabolism. Specifically, we show that DFPC1-knockdown, particularly during starvation, increases cellular free fatty acids and decreases the levels of cellular TAGs, resulting in accumulated small LDs. Using selective truncations, we demonstrate that DFCP1 accumulation on LDs in cells and *in vitro* is regulated by a previously unknown NTPase domain. Using spectroscopic approaches, we show that this NTPase domain can dimerize and can hydrolyze both ATP and GTP. Furthermore, mutations in DFCP1 that either impact nucleotide hydrolysis or dimerization result in changes in the accumulation of DFCP1 on LDs, changes in LD density and size, and colocalization of LDs to autophagosomes. Collectively, our findings suggest that DFCP1 is an NTPase that modulates the metabolism of LDs in cells.

Lipid droplets (LDs) are conserved transient lipid storage depots that can provide lipids for the repair and biogenesis of membranous organelles, as well as serving as a source of energy during times of nutrient stress. LDs accumulate in response to excessive dietary fatty acids (FAs) and can be broken down in response to meet the energy demands of the cell or the entire organism. Failure to store or catabolize lipids from LDs results in a host of metabolic diseases including lipodystrophies, obesity, insulin resistance, diabetes, NAFLD, and atherosclerosis (1). However, the molecular factors that regulate LD growth and degradation remain poorly studied.

The breakdown of LDs is a critical step in the liberation of free fatty acids (FFAs) from triacylglycerides (TAGs) stored with the LDs. This catabolism of LDs in hepatic cells requires two potentially sequential and intertwined mechanisms involving breakdown of large LDs by LD-associated lipases into small LDs, which can then be efficiently cleared by the autophagy-lysosomal pathway, in a process known as lipophagy (2, 3). During lipolysis, the protective perilipin coat surrounding LDs is removed through HSC70-dependent chaperone–mediated autophagy, thereby allowing resident lipases access to the TAGs housed within the LD core (4). Once the LD becomes sufficiently small, it can be targeted for degradation by the autophagy system, albeit this remains poorly understood (5). However, it is beginning to emerge that proteins that establish contacts between the endoplasmic reticulum (ER) and LDs are essential in facilitating the catabolism of LDs and the FA metabolism.

The ER contact site protein double FYVE domain–containing protein 1 (DFCP1) has emerged as a potential bifunctional regulator of autophagy and LD metabolism. DFCP1 (also referred to by the gene name ZFYVE1) has historically been used as a marker for the early steps of autophagy, where it is known to accumulate at phosphoinositol-3-phosphate (PI3P)-rich autophagosome precursor sites on the ER upon the induction of macroautophagy (6). This localization is due to a unique combination of structural elements that include an ER localization domain and a tandem pair of PI3P-binding FYVE domains (7). At these sites, DFCP1 was shown to mediate ring-like extensions of the ER that precede the accumulation of the autophagosome marker, LC3-II. However, in spite of this specific localization, silencing of DFCP1 does not suppress autophagosome formation during macroautophagy (6).

More recently, DFCP1 was shown to localize to LDs using a proximity assay to probe for potential interactors of the rate-limiting lipolytic enzyme, ATGL, on the LD surface (8). It has since been shown that overexpressed GFP-DFCP1 accumulates on LDs in cells, and this overexpression increases their size (9), whereas knockdown of DFCP1 was shown to do the opposite—decreasing LD size in exchange for an increase in the number of LDs. Interestingly, the function of DFCP1 on LDs may be indirect, in that it was proposed to assist in the recruitment of Seipin, as well as Rab18 and ZW10 (Centromere/kinetochore protein zw10 homolog) (9), which are two components of the NRZ ER-LD contact site complex (10). However, it should be noted that studies on the Rab18 interactome (11) and/or the purified ER-LD tethering NRZ complex (12) have not identified DFCP1 as a potential interactor. Thus, it is not clear whether DFCP1 can directly modulate LD biogenesis or whether its potential association with autophagy can be integrated with its role on LDs. For this purpose, we studied DFCP1’s localization to LDs under conditions that induce macroautophagy to determine the molecular factors that allow DFCP1 to switch between LDs and/or autophagosome precursor sites.

Here, we show that DFCP1 is an important regulator of LD metabolism. In particular, we show that DFCP1 impedes LD formation and FFA uptake and that ablating DFCP1 markedly impairs TAG accumulation while increasing FA metabolism. We demonstrate that DFCP1’s recruitment to LDs depends on a previously unreported NTPase domain and, while predicted to be a Ras-like GTPase, the NTPase domain can hydrolyze both ATP and GTP, as well as dimerize. Importantly, mutations that alter DFCP1’s NTPase activity or dimerization led to aberrant localization and accumulation of DFCP1 on LDs, as well as FA metabolism. Overall, we have established a new role for DFCP1 in FA metabolism where nucleotide-dependent association with LDs modulates FFA storage and LD size.

## Results

### DFCP1 associates with LDs during macroautophagy

**Figure 1:**
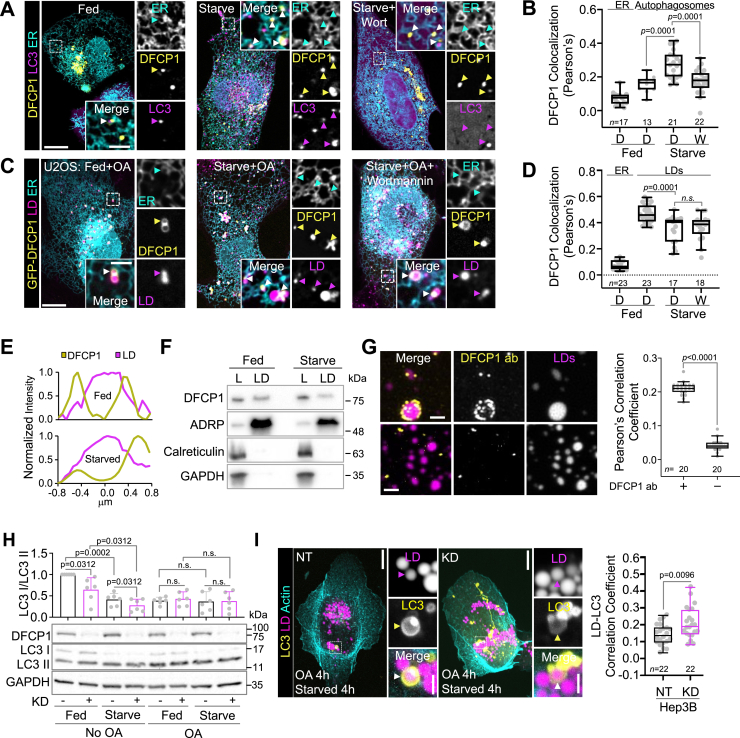
**DFCP1 localizes to LDs and autophagosomes.***A*, live cell confocal images of U2OS cells expressing mTagBFP2-DFCP1 (*yellow*), GFP-LC3 (*magenta*), and mCherry-Sec61β (*cyan*). Prior to imaging, cells were incubated 1 h in growth (*left*), starvation media (*middle*), or starvation media supplemented with 1 μM Wortmannin for 30 min (*right*). *B*, colocalization (Pearson’s correlation coefficient) of mTagBFP2-DFCP1 with the ER and LC3 from fed and starved U2OS cells treated with either DMSO (*D*) or 1 μM Wortmannin for 30 min (W). Individual colocalization measurements for each cell are plotted along with a box-and-whisker representation of the data. *C*, live cell confocal images of U2OS cells expressing mTagBFP2-DFCP1 (*yellow*) and mCherry-Sec61β (*cyan*). Cells were treated with 200 μM oleic acid (OA) for 4 h to stimulate LD formation (OA-stimulated) prior to 1 h incubations in growth media (*left*), starvation media (*middle*), or starvation media supplemented with 1 μM Wortmannin for 30 min (*right*). LipidTOX Green was used to mark LDs (*magenta*). *D*, colocalization (Pearson’s correlation coefficient) of mTagBFP2-DFCP1 with the ER and LDs from fed and starved U2OS cells treated with either DMSO (*D*) or 1 μM Wortmannin for 30 min (W). Individual colocalization measurements for each cell are plotted along with a box-and-whisker representation of the data. *E*, representative line scans across LDs from fed (*top*) and starved (*bottom*) OA-stimulated U2OS cells shown quantified in (*C*), showing DFCP1 intensity (*yellow*) relative to the LipidTOX Green intensity (*magenta*). *F*, Western blots of purified LDs isolated from fed and starved OA-stimulated U2OS cells showing the total cell lysate (L) and the purified LD fraction (LD). The LD fraction is distinguished by the marked enrichment for the LD marker ADRP and the absence of the ER marker calreticulin and cytosolic marker GAPDH. *G*, confocal images of purified LDs isolated from fed OA-stimulated U2OS cells that were stained with LipidTOX Deep Red and incubated with either anti-DFCP1 primary (*top row*) or BSA (*bottom row*) followed by an incubation with goat anti-rabbit antibody conjugated to AlexaFluor 488 secondary (scale bar represents 2 μm). The Pearson’s correlation coefficient between the endogenous DFCP1 with LDs, measured for each cell, is plotted along with a box-and-whisker representation of the data on the right. *H*, Western blot showing the conversion of endogenous LC3-I to LC3-II in clarified cell lysates from NT and KD Hep3B cells that were treated with or without 4 h OA and either fed or starved for 4 h prior to harvesting. Graph shows individual measurement of the LC3I/II ratio (determined by densitometry) along with the mean ± SD. *I*, live cell confocal images of Hep3B cells expressing LifeAct-mTagBFP2 and GFP-LC3 and stained with LipidTOX Deep Red. Cells were treated with 200 μM OA for 4 h to stimulate LD formation (OA-stimulated) prior to 1 h incubations in growth (*left*), starvation media (*middle*), or starvation media supplemented with 1 μM Wortmannin for 30 min (*right*). The Pearson’s correlation coefficient of LC3 with LDs, measured for each fed and starved Hep3B cell, is plotted along with a box-and-whisker representation of the data on the *right*. The scale bars in whole-cell and inset images represent 10 and 2 μm, respectively. The statistical significance of the colocalization measurements in panels *B*, *D*, *G*, and *I* was determined using the Mann–Whitney U-test on the indicated number of observations (indicated in each figure panel) recorded from two independent transfections. The statistical significance of the Western blot densitometry measurements in panel *H* was determined using Wilcoxon matched-pairs signed rank test from three independent transfections consisting of two replicate blots (six measurements in total). Exact *p*-values are reported with exception to *p* > 0.05, which is considered to be nonsignificant (n.s.). See also Fig. S1. BSA, bovine serum albumin; DFCP1, double FYVE domain– containing protein 1; DMSO, dimethyl sulfoxide; LD, lipid droplet.

The intracellular localization of DFCP1 is known to be sensitive to nutrient stress (6). Under normal growth conditions, DFCP1 localizes to the ER and/or the Golgi in U2OS cells (Fig. 1*A*). However, when these cells are depleted of nutrients by replacing the growth media with EBSS (hereafter referred to as starvation), they undergo a process called macroautophagy, which leads to the accumulation of DFCP1 on or near LC3-positive structures, such as autophagosomes (Fig. 1, *A* and *B*). This starvation-dependent localization of DFCP1 is known to depend on the accumulation of PI3P in subdomains of the ER important for autophagosome biogenesis, since treatment with the PI3K inhibitor Wortmannin (W) (Fig. 1, *A* and *B*), in comparison to dimethyl sulfoxide (DMSO)-treatment (D), impairs the colocalization of DFCP1 with LC3.

More recently, it has been shown that GFP-DFCP1 also localizes to LDs (8, 9, 13). Indeed, transiently expressed GFP-DFCP1 (Fig. 1, *C–E*) localizes uniformly around the periphery of LDs (Fig. 1*E*) in fed osteosarcoma (U2OS) cells that have been induced to form LDs by supplementing the growth media with 200 μM oleic acid (OA). Similarly, we also found that endogenous DFCP1 (whose localization has been previously unreported) accumulates on the periphery of LDs in fed U2OS cells (Fig. S1, *A–C*), despite having an expression that is ∼4 fold less than that of the transiently expressed GFP-DFCP1 (Fig. S1*D*). Importantly, the LD localizations of endogenous DFCP1 in fed cells are not impacted by the addition of Wortmannin (Fig. S1*A*). This localization to LDs is in addition to and independent of its ability to localize to the ER since LDs purified from OA-stimulated U2OS cells contain DFCP1 but lack the ER marker calreticulin (Figs. 1*F* and S1*E*). Notably, both endogenous DFCP1 (Fig. 1*G*) and GFP-DFCP1 (Fig. S1*F*) preserve the pattern of cellular localization on LDs isolated from cells. Thus, DFCP1 is a bona fide LD-associated protein, and transient expression of GFP-DFCP1 recapitulates the localization of endogenous DFCP1.

Given that DFCP1 can localize to LDs and autophagosomes, we investigated if starvation could influence the localization of DFCP1 to LDs. Inducing macroautophagy in the same cells leads to a modest but significant loss in GFP-DFCP1 localization to LDs (Fig. 1, *C* and *D*) and a loss of DFCP1 accumulation on LDs purified from starved cells (Fig. S1*F*). Also, in starved cells, GFP-DFCP1 appears to accumulate asymmetrically on one side of the LD (Fig. 1, *C* and *E*), typically at the junction between neighboring LDs or at the ER-LD contact site. This starvation-dependent reduction of GFP-DFCP1 on LDs persists even when starved OA-stimulated cells are treated with Wortmannin (Fig. 1, *C* and *D*), suggesting that, unlike its localization to autophagosomes, PI3P binding by DFCP1 is dispensable for its localization to LDs.

Given that DFCP1 more strongly colocalizes with autophagosomes during starvation, we wanted to explore the possibility that DFCP1 could act as a selective regulator of lipophagy. To that end, we examined the impact of DFCP1 KD on autophagosome biogenesis in hepatocellular carcinoma (Hep3B) cells where autophagy is known to play a critical role in LD metabolism (14, 15). In control Hep3B cells (cells treated with nontargeting siRNA), 4 h of starvation induced the canonical increase in the autophagosome-bound form of LC3 (LC3-II) relative to cytosolic LC3-I, and this ratio was moderately decreased in Hep3B cells (Fig. 1*H*) treated with an siRNA targeting DFCP1 (hereafter referred to as DFCP1 KD) but had no effect in U2OS cells (Fig. S1*G*). This result agrees with a similar previously published observation (6) and suggests that DFCP1 may antagonize autophagosome formation during macroautophagy. This inhibitory role of DFCP1 does not seem to be preserved in the presence of LDs since OA-stimulated control and DFCP1 KD Hep3B and U2OS cells had nearly identical ratios of LC3-I to LC3-II under both fed and starved conditions (Figs. 1*H* and S1*G*). It should be noted, however, that the addition of OA is known to stimulate autophagy (16), which gives rise to the increase in LC3-II even under fed conditions. Interestingly, while DFCP1 may not impact autophagosome formation in the presence of LDs, it does affect the targeting of LDs by autophagosomes. Both Hep3B (Fig. 1*I*) and U2OS (Fig. S1*H*) cells show improved colocalization of LC3 with LDs when DFCP1 is knocked down, indicating DFCP1 may act to inhibit LD association with autophagosomes.

### DFCP1 regulates LD catabolism and FA metabolism

**Figure 2:**
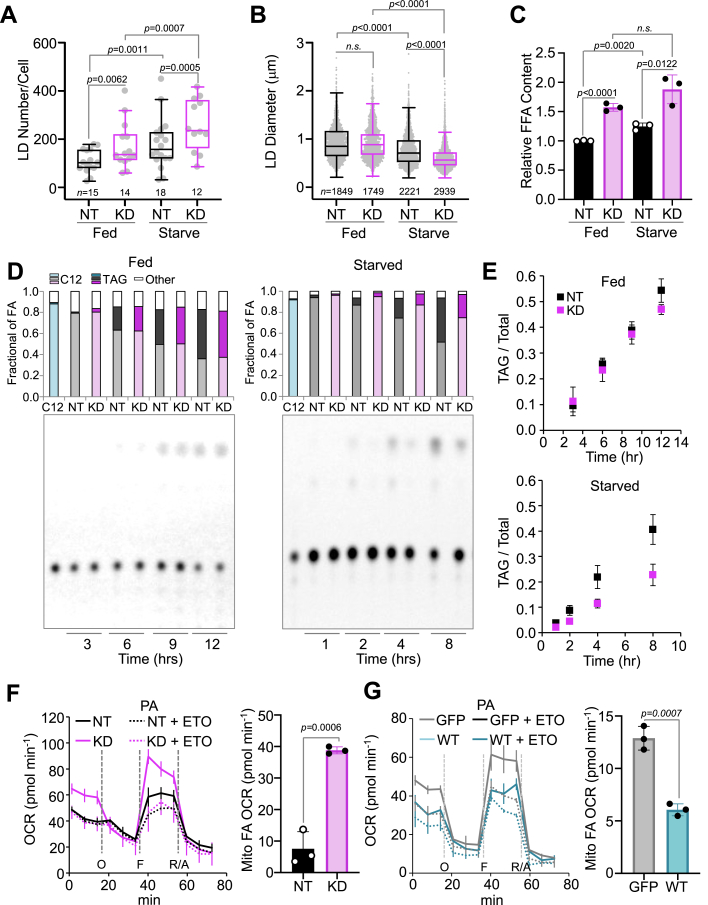
**DFCP1 regulates LD metabolism.***A* and *B*, number and diameter distributions of LDs quantified from images of control nontargeting siRNA treated (NT) and DFCP1 siRNA (KD) OA-stimulated Hep3B cells expressing LifeAct-mTagBFP2 and GFP. LDs were visualized using LipidTOX Deep Red. LD diameter was measured in the plane of a confocal Z-stack where a given LD’s diameter was the greatest. The number of LDs per cell (*A*) and the individual LD diameters (*B*) are plotted along with the box-and-whisker representations of the data. *C*, FFA content measured in NT or KD OA-stimulated Hep3B cells that were either fed or starved for 4 h. Bar graphs show individual measurements along with mean ± SD. *D* and *E*, TLC plates reporting the conversion of Bodipy C12-labeled TAGs into free Bodipy C12 FAs in control and DFCP1 KD Hep3B cells that were treated with 3 μM Bodipy C12 for 14 h (fed) or 8 h (starved). The C12 lane shows free Bodipy C12 blotted on the TLC plate immediately before mobilization. Bar graphs above the TLC plates show the fraction of free Bodipy C12 (*light cyan*, *lightgray*, and *lightmagenta*), Bodipy C12 TAGs (*dark cyan*, *darkgray*, and *darkmagenta*) and other Bodipy C12 species (*white box*). The fraction of TAG over time is plotted in *E* as mean ± SD. *F*, Seahorse assay showing the cellular oxygen consumption rate (OCR) for control (*black solid* and *dashed lines*) and DFCP1 KD Hep3B cells (*magenta solid* and *dashed lines*) stimulated with BSA-conjugated palmitic acid (PA) and in the presence of DMSO (not labeled) or Etomoxir (ETO). The OCR traces are presented as mean ± SD. The Mito FA OCR, calculated from the difference in the average mitochondria-specific uncoupled OCR (following FCCP) between DMSO and Etomoxir-treated cells, is shown as individual measurements along with mean ± SD on the right. *G*, Seahorse assay showing the cellular OCR for Hep3B cells overexpressing GFP (*gray solid* and *dashed lines*) and Hep3B cells overexpressing GFP-DFCP1 (*teal solid* and *dashed lines*) stimulated with BSA-palmitate complex and in the presence of DMSO (not labeled) or etomoxir (ETO). The OCR traces are presented as mean ± SD. Quantification of the Mito FA OCR is shown as individual measurements along with mean ± SD on the right. The statistical significance of the measurements in *A* and *B* was determined using the Mann–Whitney U-test on the indicated number of observations from two independent transfections. The statistical significance in *C* was determined using an unpaired Student’s *t* test on three independent experiments. The statistical significance in *F* and *G* was determined using a Student’s *t* test on three independent experiments recorded on the same assay plate. Exact *p*-values are reported with exception to *p* > 0.05, which is considered to be nonsignificant (n.s.). See also Fig. S2. BSA, bovine serum albumin; DFCP1, double FYVE domain– containing protein 1; DMSO, dimethyl sulfoxide; FA, fatty acid; FFA, free fatty acid; LD, lipid droplet; OA, oleic acid; TAG, triacylglyceride.

Localization of DFCP1 to LDs has previously been suggested to regulate LD growth but not biogenesis, since loss of DFCP1 drives the accumulation of LDs that are smaller on average than those found in fed WT U2OS cells (9). We also observed a similar trend in Hep3B cells. In particular, we found that DFCP1 KD led to an increase in LD number when compared to control Hep3B cells (cells treated with nontargeting siRNA), whereas the LD size distribution (as determined by the maximum diameter of each and every LD in the cell) was unaffected. Upon starvation, the number of LDs in Hep3B cells increases, but at the expense of LD diameter (Figs. 2, *A* and *B* and S2*A*). The seemingly paradoxical change in the number of LDs is likely due to the repackaging of excess FFAs liberated from LD catabolism into smaller nascent LDs (17). Furthermore, this starvation-induced accumulation of LDs is further exacerbated by DFCP1 KD, which leads to LDs that are significantly more abundant (Fig. 2*A*) and smaller (Fig. 2*B*) than those found in control Hep3B cells.

These DFCP1 KD-induced compositional changes in the LD population are also met with an increase in cellular FFAs (Fig. 2*C*), using a fluorescent-coupled acyl CoA synthetase assay that selectively labels FFAs and not TAGs. However, it is unclear if these FFAs are a result of impaired assembly of FFAs into TAGs and the subsequent packaging into LDs (biogenesis) or by accelerated lipolysis and/or lipophagy (catabolism). To better understand which of these mechanisms depend on DFCP1, we measured the incorporation of a fluorescent FA (BODIPY C12 558/568) into TAGs and the starvation-induced turnover of these fluorescent TAGs into free fluorescent FAs using TLC. In fed cells, a condition that favors LD biogenesis, we found that there was not a significant difference between control and KD cells in the incorporation of BODIPY C12 into TAGs (Fig. 2, *D* and *E*). However, in starved cells, a condition that favors LD catabolism, DFCP1 KD showed a marked reduction in TAG levels along with a concomitant increase in FFAs (Fig. 2, *D* and *E*). Since DFCP1 KD does not impact biogenesis under fed conditions, this latter result suggests that DFCP1 shields LDs from starvation-induced catabolism which would otherwise liberate FFAs. However, it should be noted that we cannot rule out a possible role for DFCP1 in regulating starvation-induced *de novo* LD biogenesis.

To further investigate the impact of DFCP1 on FA metabolism, we assessed the effect of knocking down DFCP1 on β-oxidation using a Seahorse assay. We determined the FFA-dependent oxygen consumption rate (OCR) by comparing the difference in the OCR between Hep3B cells treated with DMSO and a small molecule inhibitor (Etomoxir) for the acyl carnitine palmitoyltransferase-1 responsible for the import of FFAs into mitochondria. In this assay, control Hep3B cells showed little difference in the basal (prior to oligomycin treatment) and maximal respiratory capacity (following uncoupling by FCCP) when treated with DMSO or Etomoxir (Fig. 2, *F* and *G*). By contrast, DFCP1 KD Hep3B cells showed a nearly 2-fold increase in the total uncoupled OCR and nearly 4-fold increase in the mitochondria-specific uncoupled OCR (the difference in nonmitochondrial OCR following rotenone and antimycin A treatment from the uncoupled OCR following FCCP treatment) when compared to the same cells treated with Etomoxir (Fig. 2, *F* and *G*). Supplying additional saturated (palmitate) and unsaturated (oleate) FAs (Figs. 2*F* and S2*B*, respectively) increased the maximum OCR for both control and KD cells, with KD cells showing a greater increase in OCR for both FAs. While this increase in FFA-dependent OCR by DFCP1 KD could be reversed with overexpression or rescue with GFP-DFCP1, but not GFP (Figs. 2*G* and S2*C*), such overexpression also led to a marked suppression of both the basal and uncoupled OCR in both control and KD cells (Figs. 2*G* and S2*C*). When taken together, these data support a mechanism whereby DFCP1 impairs the availability of FFAs for FA-dependent mitochondrial respiration.

### DFCP1 localizes to LDs using two distinct structural domains

**Figure 3:**
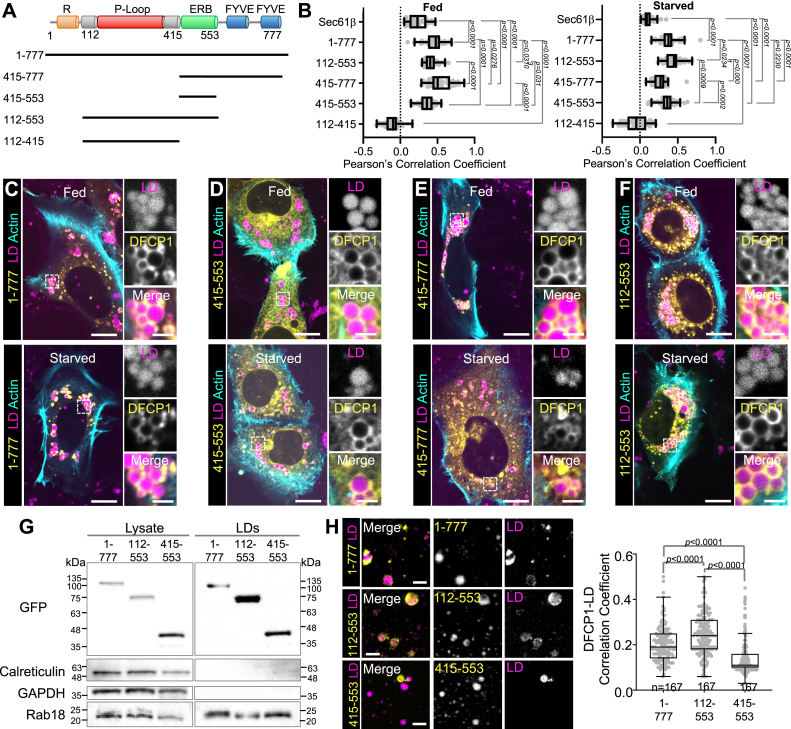
**Domain requirements for DFCP1 localization to LDs.***A*, domain diagram of DFCP1 depicting the N-terminal Ring-like domain (R), the NTPase domain (P-loop), the endoplasmic reticulum–binding domain (ERB), and the tandem FYVE domains (FYVE). GFP-tagged constructs used in this figure are depicted below the domain diagram. *B*, the extent of colocalization (Pearson’s correlation coefficient) between GFP-DFCP1 and LDs from fed (*left*) and starved (*right*) cell populations depicted in panels *C–F*. The extent of colocalization between GFP-Sec61β and LDs is also included as a reference (Fig. S3*C*). Individual colocalization measurements from each cell are plotted along with a box-and-whisker representation of the data. *C–F*, representative images of U2OS cells expressing LifeAct-mTagBFP2 (*cyan*) and the GFP-DFCP1 truncations (*yellow*) indicated in *A*. Prior to imaging, all cells were treated with 200 μM OA for 20 h before incubating in either growth or starvation media for 18 h. LDs were visualized with LipidTOX Deep Red (*magenta*). *G*, Western blots of clarified lysates (*left*) and isolated LD fraction (*right*) from OA-stimulated DFCP1 KO U2OS cells rescued with GFP-DFCP1 constructs 1-777, 112-553, and 415-553. The LD fraction is distinguished by the absence of calreticulin and GAPDH. Rab18 was used as a load control since its abundance does not depend on the accumulation of DFCP1 on LDs. *H*, confocal images of isolated LDs in (*G*) and labeled with LipidTOX Deep Red. The Pearson’s correlation coefficient between the indicated GFP-DFCP1 constructs with LDs, measured for each cell, is plotted along with a box-and-whisker representation of the data on the *right*. The scale bars in whole-cell and inset images represent 10 and 2 μm, respectively. The statistical significance of the measurements in panels *B* and *H* was determined using the Mann–Whitney U-test. Exact *p*-values are reported with exception to those that are >0.05, which are considered to be nonsignificant (n.s.). See also Fig. S3. DFCP1, double FYVE domain–containing protein 1; ER, endoplasmic reticulum; LD, lipid droplet; OA, oleic acid.

In the presence of LDs, DFCP1 is less sensitive to this starvation-induced translocation to sites of autophagosome biogenesis on the ER. This suggests that additional intramolecular factors counteract PI3P-mediated transmigration of DFCP1 to sites of autophagosome formation. To identify these additional domains that may contribute to DFCP1’s localization to LDs, we transiently expressed GFP-DFCP1 truncations (Figs. 3 and S3) in fed and starved OA-treated U2OS cells. The localization of DFCP1 to either the ER or LDs is known to require the endoplasmic reticulum–binding domain motif (ERB) (6, 9, 13), and starvation-induced translocation to PI3P-rich regions is known to also require the C-terminal tandem FYVE domains (6, 18). In addition to the FYVE domains and the ERB motif, DFCP1 has an N-terminal domain that bears some similarity to a ring-domain (R) and a folded domain containing a canonical P-loop sequence (P-loop) (Fig. 3*A*). Among all the GFP-constructs tested, those constructs containing the ERB (1-777, 112-553, 415-553, 415-777, 112-777, 1-553) show greater LD localization (as determined by Pearson’s correlation coefficient) under fed and starved conditions (Figs. 3, *B*–*F* and S3, *D*, *E* and *H*), when compared to the promiscuous ER marker, Sec61β (Figs. 3*B* and S3, *C* and *H*). By contrast, constructs lacking the ERB (Fig. S3, *F* and *G*) are completely cytosolic in both fed and starved cells and therefore show negative colocalization with LDs (Figs. 3*B* and S3*H*). Importantly, this is not a result of excessive transient expression, as constructs lacking the ERB display more attenuated expression than those that do contain the ERB (Fig. S3*B*).

While the ERB is essential to localize DFCP1 to LDs, it does not, however, confer sensitivity to starvation. The ERB domain construct (415-553) localizes to both the ER and LDs in both fed and starved cells, and this localization is not as strong as those constructs that also contain a P-loop domain (Fig. 3*B*). By contrast, a construct that contains both the ERB and the FYVE domains (415-777) localizes as well as full-length DFCP1 to LDs in fed cells but is markedly less localized to LDs in starved cells (Fig. 3, *B* and *E*). Notably, this colocalization to LDs in starved cells is the least of all other constructs that contain an ERB (Figs. 3*B* and S3*H*). By contrast, those constructs containing the P-loop domain and the ERB (1-777, 112-777, 1-553, and 112-553) stay strongly localized to LDs during starvation (Figs. 3, *C* and *F* and S3, *D* and *E*), countering the starvation-induced loss of LD localization seen in constructs containing the FYVE domains. In support of this observation, GFP-DFCP1 112-553 is more enriched in the LD fraction isolated from rescued DFCP1 CRISPR-KO U2OS cells than LD fractions from cells rescued with the full length and the ERB constructs (Fig. 3*G*). Coincidently, this construct is also more colocalized with LDs *in vitro* (Fig. 3*H*). Thus, this truncation analysis reveals that the P-loop domain plays a role in the localization and/or stability of DFCP1 on LDs during starvation.

### DFCP1 is a novel P-type NTPase

**Figure 4:**
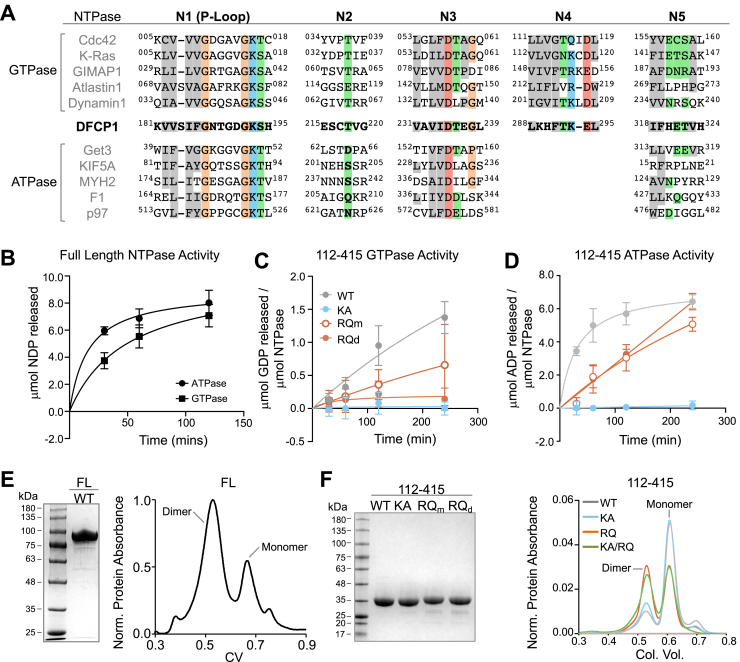
**Characterization of the DFCP1 NTPase domain.***A*, sequence alignment of the human DFCP1 N-box motifs with several representative human GTPases and ATPases. *B*, ADP (*circle*) and GDP (*square*) release assays using 20 μM ATP or GTP, respectively, and 2 μM of the FL DFCP1. Plotted data points represent mean ±SD for three independent experiments. *C*, GDP release assay using 20 μM GTP and 2 μM of the following DFCP1 constructs: WT (112-415; *gray*), KA (112-415 containing the K193A mutation; *blue*), or RQ_m_/RQ_d_ (monomeric/dimeric states of 112-415 containing the R266Q mutation; *orange open* and *closed*). Plotted data points represent mean ±SD for three independent experiments. *D*, ADP release assay using 20 μM ATP and 2 μM of the DFCP1 truncations in *C*. Plotted data points represent mean ±SD for three independent experiments. *E*, SDS-PAGE gel and size-exclusion chromatography profile of full-length FLAG-DFCP1. *F*, SDS-PAGE gel and size-exclusion chromatography profile of WT (*gray*), KA (*blue*), RQ (*orange*), and KARQ (construct that contains both the K193A and R266Q mutations; *green*) MBP-DFCP1 (112-415). See also Fig. S4. DFCP1, double FYVE domain–containing protein 1.

To date, the significance of the phosphate binding loop (P-loop) in DFCP1 has not been determined. This is surprising given that the P-loop domain is an essential motif of P-type NTPases and is also a highly conserved feature of DFCP1 found across all species (Fig. S4*A*). This prompted us to identify other motifs in DFCP1 that are commonly associated with nucleotide hydrolysis in bona fide P-type NTPases (Fig. 4*A*). NTPases contain either four or five essential sequence motifs, called N-boxes, that are critical for the hydrolysis of ATP or GTP, respectively (19). Among these, only N1 (P-loop) and N3 are the most conserved among both P-type ATPases and GTPases, whereas N2 and N5 are poorly conserved. The fourth N-box (N4) is unique to and conserved among GTPases. The presence of the N4 motif strongly suggests that DFCP1 NTPase domain preferentially hydrolyzes GTP. Furthermore, the amino acid separation between N-box elements N1 and N4 is also well conserved within the family of NTPases (Fig. S4*B*), with Ras superfamily NTPases having the shortest amino acid separation (∼100 residues) while Gαs have the largest separation (∼225 residues).

To confirm that DFCP1 possesses a bona fide NTPase domain, we expressed and purified full-length and NTPase-domain truncations of DFCP1 and determined the amount of ADP or GDP formed over time by measuring the competitive displacement of a fluorescent GDP molecule from a fluorescence-quenching GDP antibody. Using this assay, we found that full-length DFCP1 (FL), purified from mammalian cells, had almost equivalent turnover rate for both ATP and GTP (Fig. 4, *B*–*D*). Interestingly, the GTP turnover rate of ∼0.03 μmol GDP μmol NTPase^−1^ min^−1^ is similar to the basal rate of GTP hydrolysis by Dynamin I (∼0.04 μmol GDP per μmol NTPase per min) measured using the same assay (20). The isolated NTPase domain also had an ATPase rate similar to the full-length protein, however, the GTP rate was considerably attenuated (∼0.004 μmol GDP per μmol NTPase^−^ per min) and is on the same order of the basal activity of a Ras GTPase, like Cdc42 (Fig. S4*C*). Importantly, both the ATPase and GTPase activity of this domain was abolished by mutating the highly conserved lysine in the P-loop (K193) to an alanine (Fig. 4, *C* and *D*), which is commonly done to disrupt nucleotide binding in bona fide NTPases (21, 22, 23, 24), as well as by an excess of magnesium chelating agents such as EDTA or through a high concentration of phosphate (Fig. S4*C*).

A common feature of NTPases is the ability to undergo nucleotide-mediated dimerization, which often helps to enhance membrane-binding of these NTPases as well as their activities on membrane surfaces (25, 26). We therefore assessed the oligomerization state of DFCP1 using size-exclusion chromatography. Both the full-length DFCP1 (Fig. 4*E*) and the purified NTPase domain (Fig. 4*F*) exists in an equilibrium between monomeric and dimeric states, with full-length DFCP1 favoring a dimeric state and the isolated NTPase domain strongly favoring the monomeric state. The latter equilibrium is not perturbed by nucleotide binding as the K193A mutation reproduces the monomer-dimer equilibrium of the WT NTPase domain (Fig. 4*F*).

To further probe the relationship between nucleotide activity and dimerization, we introduced a previously unstudied cancer-related mutation into DFCP1. Among all the residues of DFCP1, only the R266Q mutation has been found prevalently in patients (Fig. S4*D*). Curiously, this mutation was found to occur in patients with either endometrial and colorectal cancers, which are two cancers distinguished by the presence of an abnormal abundance of LDs (27). We therefore wondered if this mutation could serve as a vital link between DFCP1’s biochemical properties and its role on LDs. Introducing this mutation into the NTPase domain resulted in a marked accumulation of the dimeric NTPase domain (Fig. 4*F*). Additionally, both the monomeric and dimeric states resulting from this mutation showed reduced ATPase and GTPase activity (Fig. 4, *C* and *D*, respectively), although not nearly to the same extent as the K193A mutation. We therefore hypothesized that DFCP1 may function like several other NTPases, where NTP binding mediates dimerization and monomerization is coupled to nucleotide hydrolysis and/or release. To address this question, we examined the oligomerization potential of a DFCP1 NTPase domain construct carrying both the K193A and R266Q mutations (KA/RQ). This construct, which lacks the ability to bind to either ATP or GTP, was still able to dimerize to the same extent as R266Q alone (Fig. 4*F*). Thus, dimerization mediated by R266Q is independent of nucleotide binding.

### DFCP1’s NTPase activity regulates LD metabolism

**Figure 5:**
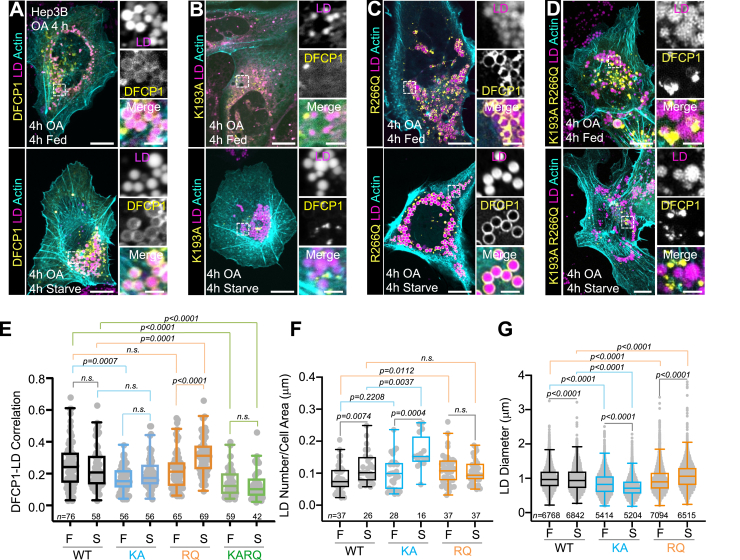
**The NTPase activity of DFCP1 modulates LD accumulation.***A–D*, live-cell confocal images of DFCP1 KD Hep3B cells rescued with either WT (WT, *A*), K193A (KA, B), R266Q (RQ, *C*), and K193A/R266Q (KARQ, *D*) GFP-DFCP1 constructs (*yellow*) and treated with LipidTOX Deep Red (*magenta*). Prior to imaging, cells were OA-stimulated for 4 h before incubating in either growth or starvation media for 4 h. *E*, the extent of colocalization (Pearson’s correlation coefficient) between WT (*black*) and GFP-DFCP1 mutants KA (*blue*), RQ (*orange*), and KARQ (*green*) with LDs, measured from each cell of the cell populations depicted in Figure 4, *A–D*, is plotted along with a box-and-whisker representation of the data on the *right*. *F* and *G*, density and diameter distributions of LDs quantified from images represented in *A–C*. LD densities were determined for each cell by dividing the total number of LDs for a given cell by the cell’s area. The individual densities along with a box-and-whisker representation of the data are plotted in *F*. The LD diameter (visualized using LipidTOX Deep Red) was measured in in the plane where a given LD’s diameter was the largest. Each LD diameter along with a box-and-whisker representation of the data is plotted in *G*. The statistical significance of the measurements in panels *E–G* was determined using the Mann–Whitney U-test based on the number of observations indicated in each figure panel, which were recorded from two independent transfections. Exact *p*-values are reported with exception to those that are >0.05, which are considered to be nonsignificant (n.s.) The scale bars in whole-cell and inset images represent 10 and 2 μm, respectively. See also Fig. S5. DFCP1, double FYVE domain–containing protein 1; LD, lipid droplet; OA, oleic acid.

To determine the impact of the DFCP1 NTPase domain on LD metabolism, we transiently rescued Hep3B DFCP1 KD cells with full-length GFP-DFCP1 constructs bearing either single residue mutations (K193A and R266Q) or a combination of the two (K193A/R266Q) in the NTPase domain. In fed and starved OA-treated Hep3B cells, WT GFP-DFCP1 uniformly coats LDs (Fig. 5, *A* and *E*). By contrast, GFP-DFCP1 K193A (KA) localizes poorly to LDs under both nutrient conditions and instead forms puncta that remain well-separated from LDs (Fig. 5, *B* and *E*). The R266Q mutation (RQ), on the other hand, shows a similar accumulation on LDs as WT DFCP1 under fed conditions, but this accumulation is markedly increased upon starvation (Fig. 5, *C* and *E*). These single point mutations suggest that both nucleotide binding and dimerization promote localization to LDs, but it is unclear which of these features are essential since the R266Q mutation moderately impairs the NTPase activity (Fig. 4*C*). To address this question, we examined the localization of GFP-DFCP1 KA/RQ, which prevents nucleotide binding while facilitating dimerization. Unlike either WT or the RQ mutation, the KA/RQ mutant was less localized to LDs in both fed and starved cells and to an extent similar to that of the KA construct (Fig. 5, *D* and *E*). Notably, these differences in DFCP1 accumulation on LDs are not a result of expression differences in these constructs (Fig. S5*A*). Thus, nucleotide binding and not dimerization is necessary for the assembly of DFCP1 on LDs.

Given that LD size and density correlates with the expression of DFCP1 (Fig. 2, *A* and *B*), and that the DFCP1 NTPase domain is important for its localization to LDs (Fig. 5, *A*–*D*), we hypothesized that NTPase mutations could potentiate LD turnover. Under fed conditions, Hep3B DFCP1 KD cells rescued with either NTPase mutations are able to generate equivalent numbers of LDs (Fig. 5*F*) as WT rescued cells, but the size distribution of these LDs differ significantly between each construct (Fig. 5*G*). Rescuing with the K193A mutant causes LDs to be significantly smaller than those found in the WT rescue cells, correlating with a decrease of DFCP1 K193A on the LDs (Fig. 5*E*). Upon starvation, the number of LDs in WT and K193A rescued cells increased, with the number of LDs in the starved K193A rescue cells being significantly more than the number formed in WT. This change in LD number is anticorrelated with the size of LDs, as the K193A rescue cells have LDs that are considerably smaller than those found in WT rescue cells, replicating the results seen in our DFCP1 KD. By contrast, cells rescued with R266Q mutation do not show a significant change in the density of LDs but have LDs that are considerably larger than those found in WT cells.

Consistent with these changes in LD size, there are correlated changes in FFA metabolism. Overexpression of constructs that bind more tightly to LDs (WT and RQ) suppressed the FFA-dependent portion of the uncoupled OCR in control Hep3B cells, whereas the construct that does not effectively localize to LDs (KA) had little impact on the FFA-dependent OCR (Fig. S5*B*). This suggests that DFCP1 accumulation on LDs impedes FA flux into mitochondria, albeit this mechanism remains unclear. Altogether, this suggests that the LD size distribution and availability of FFAs depends on the ability of DFCP1 to coat the LDs and, by extension, the nucleotide-bound state of DFCP1.

### DFCP1’s NTPase activity switches its localization from LDs to autophagosomes

**Figure 6:**
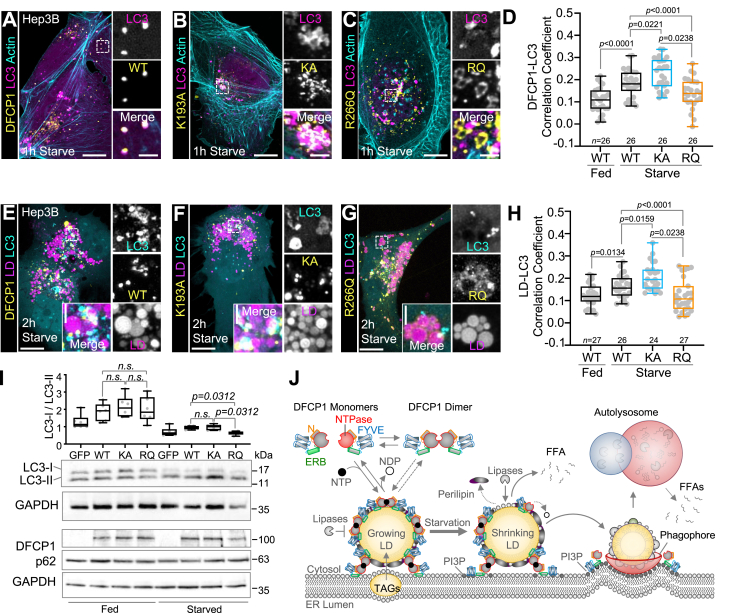
**DFCP1 mutations modulate localization of autophagosome targeting of LDs.***A–C*, live-cell confocal images DFCP1 KD Hep3B cells expressing LifeAct-mTagBFP2, mCherry-LC3, and either WT (WT, *A*), K193A (KA, *B*), or R266Q (RQ, *C*) GFP-DFCP1 constructs. Prior to imaging, cells were incubated in either growth or starvation media for 1 h. *D*, the extent of colocalization (Pearson’s correlation coefficient) between GFP-DFCP1 and mCherry-LC3, measured from each cell for the cell populations depicted in Figure 5, *A*–*C*, is plotted along with a box-and-whisker representation of the data on the right. *E–G*, live-cell confocal images of DFCP1 KD Hep3B cells treated with LipidTOX Deep Red and expressing mCherry-LC3 and either WT (*E*), K193A (*F*), or R266Q (*G*) GFP-DFCP1 constructs. Prior to imaging, cells were treated with OA for 4 h before incubating in either growth or starvation media for 1 h. *H*, the extent of colocalization (Pearson’s correlation coefficient) between GFP-LC3 and LDs, measured in each cell for the cell populations depicted in Figure 5, *D*–*F*, is plotted along with a box-and-whisker representation of the data on the *right*. *I*, Western blot showing the abundance of p62 and the conversion of endogenous LC3-I to LC3-II in clarified cell lysates from rescued DFCP1 KD Hep3B cells that were treated with OA for 4 h and either fed or starved for 4 h prior to harvesting. Graph shows individual measurement of the LC3I/II ratio (determined by densitometry) along with the mean ± SD. *J*, model of the role of DFCP1 in LD metabolism. Nucleotide binding to DFCP1 facilitates localization of DFCP1 to LDs, which is stabilized through oligomerization. DFCP1 accumulation on LDs ultimately inhibits the lipolytic breakdown of LDs during starvation and as a consequence, the targeting of LDs by autophagosomes. The scale bars in whole-cell and inset images represent 10 and 2 μm, respectively. The statistical significance of the measurements in panels *D* and *H* was determined using the Mann–Whitney U-test based on the number of observations indicated in each figure panel, which were recorded from two independent transfections. The statistical significance of the Western blot densitometry measurements in panel *I* was determined using Wilcoxon matched-pairs signed rank test from three independent transfections consisting of two replicate blots (six measurements in total). Exact *p*-values are reported with exception to *p* > 0.05, which are considered to be nonsignificant (n.s.). DFCP1, double FYVE domain–containing protein 1; LD, lipid droplet; OA, oleic acid.

Under conditions of macroautophagy, DFCP1 mobilizes to sites of autophagosome biogenesis on the ER (Fig. 1*A*). We therefore wanted to assess whether mutations in the DFCP1 NTPase domain could also modulate the ability of DFCP1 to colocalize with autophagosomes in starved and rescued DFCP1 KD Hep3B cells. As previously observed for HEK293 cells (6), starvation in the absence of LD stimulation leads to an increase in DFCP1 puncta that colocalize preferentially with LC3 puncta in Hep3B cells (Fig. 6, *A* and *D*). This colocalization was further enhanced with the K193A mutation, which mainly forms small puncta that decorate LC3 clusters (Fig. 6, *B* and *D*). In contrast, the R266Q mutation leads to the formation of ring-like tubular structures, which were well separated from LC3 puncta (Fig. 6, *C* and *D*).

DFCP1 mutations also influence the localization of LC3 to LDs. As noted above, LC3 becomes enriched around LDs during amino acid and FFA starvation, particularly in DFCP1 KD cells (Fig. 1*I*). Consistent with this observation, DFCP1 KD Hep3B cells rescued with WT DFCP1 show a starvation-dependent enrichment of autophagosomes on LDs (Fig. 6, *E* and *H*). This colocalization is significantly improved in cells rescued with the K193A mutation (Fig. 6, *F* and *H*) but considerably diminished in cells rescued with the R266Q mutation (Fig. 6, *G* and *H*). However, despite these changes in autophagosome-LD colocalization, DFCP1 NTPase mutants exerted mild effects on autophagy flux. In fed cells, the ratio of LC3-I to LC3-II was not significantly different between cells rescued either with WT or mutant GFP-DFCP1. Under starved conditions, we only observe a modest, albeit significant, inhibition of LC3 processing in cells rescued with the K193A mutation when compared to the R266Q mutation. In contrast to the mild changes observed with LC3, we did not observe any appreciable difference in the abundance of the ubiquitous autophagy adaptor protein, P62/SQSTM1 (Fig. 6*I*). Altogether, this data demonstrates that the ability for DFCP1 to localize to either LDs or autophagosomes is dependent on DFCP1’s NTPase activity, and LD localization is anticorrelated with its localization to autophagosomes.

## Discussion

LDs are transient energy storage depots that help to buffer cellular energy demands. As a consequence, LDs can rapidly form when FAs are abundant but are also quickly brokendown when ATP becomes scarce. While the physiological roles of LDs are well established, the molecular regulation of LDs remains poorly understood. In this study, we expand on emerging reports that the autophagy-associated protein DFCP1 is a regulator of LD metabolism. Specifically, we show that DFCP1 reciprocally accumulates on LDs and sites of autophagosome formation (Fig. 1), depending on the nutrient status of the cell. DFCP1 can modulate FA metabolism as well as TAG breakdown, and knocking down DFCP1 results in altered LD sizes and numbers (Fig. 2). This accumulation on LDs is controlled by a previously uncharacterized NTPase domain that works in conjunction with the ER-binding motif to regulate its association to the periphery of LDs (Fig. 3). This NTPase domain is unique in that it possesses the ability to hydrolyze both ATP and GTP as well as the ability to dimerize (Fig. 4). In cells, modulating the function of the NTPase domain through mutagenesis leads to aberrant accumulation of DFCP1 on LDs and a change in LD metabolism (Fig. 5). Coincidentally, these same mutations also modulate the colocalization of LDs with autophagosomes. Specifically, impairing nucleotide binding leads to loss of DFCP1 on LDs and increased association of LDs with autophagosomes, whereas a mutation-driving dimerization of DFCP1 increases stability of DFCP1 on LDs and impairs the autophagosome-LD association (Fig. 6). Collectively, our data suggests a mechanism where DFCP1 can bind LDs and to repress FA-dependent metabolism, thereby resulting in an increase of TAG levels. The presence of DFCP1 at PI3P-rich regions instead of on LDs, as demonstrated by the KA mutation, appears to cause an increase in association of LDs with LC3 along with a mild inhibition of autophagy efficiency which correlates with an increase in small LDs. This suggests that the accumulation of DFCP1 at PI3P-rich regions instead of the LDs promotes lipolysis of larger LDs while simultaneously repressing lipophagy, thereby causing small LDs to accumulate. In this way, DFCP1 functions to regulate FA metabolism in response to nutrient availability (Fig. 6*J*).

While the impact of DFCP1 on LD metabolism is clear, the specific phase of LD breakdown regulated by DFCP1 remains uncertain. Recently, it has been suggested that LDs follow a two-step catabolic pathway. First, larger LDs are reduced in size through the combined actions of perilipin removal by chaperone-mediated autophagy and the breakdown of TAGs housed within the LD by LD-associated lipases (4). This process, which is thought to require declustering and/or a separation of LDs from the ER (28), ultimately results in LDs with reduced volume. When LDs become sufficiently small, they can be consumed in whole by the canonical autophagy system (5). Consequently, impairing lipolysis leads to a net increase in the number of larger LDs, since smaller LDs would be preferentially cleared by the autophagy system; whereas, impairing autophagy is expected to result in an accumulation of small LDs that have been processed from large LDs undergoing lipolysis (5). The seemingly paradoxical change in the number of LDs is likely due to the repackaging of excess FFAs liberated from LD catabolism into smaller nascent LDs (17). We have observed that knockdown of DFCP1 leads to changes in the LD size distribution that is consistent with inhibition of autophagy (Fig. 2, *A* and *B*); however, neither knockdown of DFCP1 nor rescuing with DFCP1 NTPase mutants had a marked effect on autophagosome formation (Figs. 1*H*, S1*G* and 6*I*). Alternatively, it is possible that DFCP1 slows down the rate of lipolysis and thereby give more time for the autophagy system to clear the small LDs and prevent their accumulation. Consistent with this hypothesis, we only observed a significant dependence on DFCP1 on the rate of TAG lipogenesis in fed but not starved cells (Fig. 2, *D* and *E*). The latter is indicative of an increase in the rate of LD turnover that counteracts lipogenesis during starvation. This suggests that DFCP1 might also regulate steps of LD catabolism upstream of lipophagy, such as lipolysis, although more work is needed to characterize the exact mechanism by which DFCP1 regulates FA metabolism.

Large GTPases, but not ATPases, are also known to be involved in modulating contacts between the ER and other organelles. For example, Mitofusin 2 tethers and regulates the spacing between the outer mitochondrial membrane to the ER (29). Similarly, the dynamin-like Atlastins were shown to regulate tethering of COP II–coated vesicles to the ER (30). In both of these cases, tethering is driven by GTP-dependent oligomerization of the GTPase domain and multivalent membrane binding to the ER and another organelle. DFCP1 shares the same basic molecular architecture of these large GTPases, including the ability to dimerize and the capacity to bind to multiple membrane compartments. Interestingly, our initial sequence prediction for DFCP1 revealed that its P-loop bears similarity to the P-loop found in the GTPases of Immunity Associated Proteins (GIMAPs), which are a class of septin-like NTPases that have been shown to regulate LD metabolism (31) and interact with LDs in immune cells (32) For example, GIMAP2 has been found to localize directly to LDs, using its dual hydrophobic C-terminal domain to associate and potentially tether LDs to each other (33), and it's overexpression leads to an increase in the number of LDs. GIMAPs have not been shown to regulate LDs outside lymphocytes, and therefore it is reasonable to speculate whether DFCP1 may replace GIMAPs in other cell types. However, aside from the P-loop, DFCP1 bears little sequence or structural similarity to GIMAP proteins, which may be a result of specific sequence adaptations needed to hydrolyze ATP. Clearly, future structural and kinetic studies will be important to reveal the precise mechanism of ATP and GTP binding.

While many large GTPases have been implicated in establishing membrane contact sites, DFCP1 was only shown to establish an ER-LD contact site through the formation of a tethering complex consisting of NAG-RINT1-ZW10 (NRZ) and Rab18 (9). In doing so, this complex is able to bring LDs in close proximity to the ER in order to drive LD expansion during biogenesis (9). During macroautophagy, the NRZ complex and its components are known to take on more passive roles, such as tethering components needed for autophagy initiation at the ER or the mobilization of ATG9 out of the TGN (34). By contrast, DFCP1 is well established to accumulate on PI3P-rich regions on the ER that frequently associated with the sites of autophagosome biogenesis. This suggests that DFCP1 may also function as a tethering factor that ensures that LDs are tethered to the ER near sites of autophagosome formation, even when the NRZ complex is absent. Additionally, this binding to PI3P could also serve to sequester DFCP1 that has undergone GTP-dependent disassembly from LD surface. In this way, DFCP1 could promote tethering of LDs near degradative subdomains of the ER, which would then be released for rapid catabolism in response to an environmental trigger, although further studies will be necessary to elucidate the specific role that DFCP1 plays in the LD lifecycle.

In summary, we have shown that DFCP1 is a regulator of LD metabolism. Specifically, we show that DFCP1 is a novel NTPase that accumulates on LDs in fed cells and in a nucleotide-dependent manner. However, during starvation, DFCP1 partially redistributes away from LDs onto the ER at PI3P-rich regions to regulate LD catabolism. Thus, it is tempting to speculate that DFCP1 functions as a molecular switch that promotes LD growth under basal conditions and LD catabolism during starvation.

## Experimental procedures

### Mammalian cell culture

Hep3B and U2OS cells were cultured at a 37 °C with 5% CO_2_ and in growth media consisting of MEM GlutaMax or Dulbecco’s modified Eagle’s medium (DMEM) GlutaMax supplemented with 10% fetal bovine serum (FBS) and antibiotic-antimycotic (Thermo Fisher Scientific), respectively. Two days prior to live-cell imaging experiments, 50,000 cells were seeded on 3.5 cm imaging dishes and grown to 40% confluency prior to transfection with the indicated plasmids using FuGENE HD (Promega). In some cases, cells were also incubated for 2 days before imaging with a siRNA mixture, consisting of 5 μl of RNAiMax (Thermo Fisher Scientific) and 2.5 pmol of the specified siRNA. Cells were induced to form LDs by supplementing the growth media with 200 μM OA (dissolved in ethanol) for 4 or 24 h (as indicated), before exchanging the media with either normal growth media or EBSS for 1, 4, or 18 h (as indicated) prior to imaging. LDs were identified by incubating with the LD-specific dyes Bodipy-C12-568 (Thermo Fisher Scientific), LipidTox Green (Thermo Fisher Scientific), or LipidTox Deep Red (Thermo Fisher Scientific) at volume ration of 1:10,000 for 30 min prior to imaging. In some cases, cells were treated with 1 μM Wortmannin (Cayman Chemical Company) for 30 min prior to imaging.

### U2OS DFCP1 knock-out cell line

The U2OS DFCP1 knock-out cell line was generated using Alt-R CRISPR/Cas9 system (IDT). A custom guide RNA (UAGCAGUGACGAUACGGAAGUUUUAGAGCUAUGCU) was annealed to tracer RNA and incubated with purified Streptococcus pyogenes Cas9 nuclease (IDT) to form Cas9-guide RNA complexes. These complexes were electroporated into U2OS cells using an Amaxa 4D-Nucleofector (Lonza Biosciences). Electroporated cells were allowed to recover for 72 h, before single-cell sorting into 96-well plates. Cells were allowed to reach confluency before they were harvested and checked for DFCP1 expression using Western blotting. Those clonal populations where DFCP1 was ablated were further analyzed by next generation sequencing using the CRISPR guide as a sequencing primer.

### Live cell imaging and image analysis

All cells were imaged using either a Nikon Ti2 inverted microscope equipped with a 100 × (1.4 NA) Plan-Apo oil immersion objective and a Yokogawa CSU-W1 spinning disk confocal attached to a Hamamatsu ORCA-FLASH4.0 CMOS camera. Cells in either DMEM FluoroBrite (Thermo Fisher Scientific) medium supplemented with 5% FBS or EBSS lacking phenol red (MilliporeSigma) were imaged at 37 °C and 5% CO_2_. Images stacks were captured at 16-bit 2048 × 2044 resolution with an axial spacing of 0.2 μm using the Nikon Elements Software package. All images were captured blindly and randomly, which involved imaging the nearest cell to a random set of x-y coordinates that contained actin fluorescence (depending on the experiment). Captured images were blinded again, and image analysis was performed using the software Fiji (https://imagej.net/Fiji). Specifically, LD number and diameters were scored manually, and 2D/3D colocalization analysis was performed using the Fiji Coloc2 analysis tool. Specifically, LD diameter was measured in the plane where a given LD’s diameter was the largest and LD density was determined by dividing the total number of LDs by the area of the cell’s basement membrane.

### GDP release assay

All GTPase assays were performed with the GDP-FI Transcreener assay (Bellbrook Labs) according to the manufacturer’s suggested protocol. In brief, GTPase constructs were added to a tube containing GDP Release Assay buffer (3 mM MgCl_2,_ 1 mM DTT, 150 mM NaCl, 20 mM Tris–HCl, pH 8.0) and 20 μM GTP and incubated at room temperature in 25 μl reactions. Reactions were quenched by the addition of 25 μl stop solution (18.8 μg/ml Ab-IR dye QC-1, GDP-AlexaFluor 594 tracer, and 1× Stop and Detect solution) and immediately read using a plate reader for 1 h, taking measurements every 10 min.

### ADP release assay

All ATPase assays were performed with the GDP-FI Transcreener assay (Bellbrook Labs) according to the manufacturer’s suggested protocol. In brief, ATPase constructs were added to a tube containing ADP Release Assay buffer (3 mM MgCl_2,_ 1 mM DTT, 150 mM NaCl, 20 mM Tris–HCl, pH 8.0) and 20 μM ATP and incubated at room temperature in 25 μl reactions. Reactions were quenched by the addition of 25 μl stop solution (18.8 μg/ml Ab-IR dye QC-1, GDP-AlexaFluor 594 tracer, and 1× Stop and Detect solution) and immediately read using a plate reader for 1 h, taking measurements every 10 min.

### FFA uptake and content assays

To assess FFA uptake, DFCP1 KD Hep3B cells rescued with the indicated DFCP1 constructs and seeded in a 96-well polylysine-coated tissue culture plate. Cells were serum deprived for 1 h before adding the fluorescent TF2-C12 Fatty Acid (MilliporeSigma). Fluorescence emission was monitored for 1 h at 37 °C on a Citation 5 microplate reader (BioTek), with the excitation monochromator set to 485 ±7 nm and the emission monochromator set to 515 ±7 nm. FFA concentrations from Hep3B cells were measured with Free Fatty Acid Quantitation Kit reagents according to the manufacturer's instructions (MilliporeSigma).

### TLC assay

Cells were seeded on 10 cm dishes on Day 0 and then treated with RNAImax and siRNA or scr on Day 1. On Day 3, cells were washed with PBS and incubated with 3 μM BODIPY-C12 558/568 in EBSS (starved) or MEM with 10% FBS (fed) in a time-dependent manner. Cells were harvested *via* scraping and spun down in PBS. Cellular lipids were extracted in chloroform and spotted on aluminum-backed silica plates (Sigma) and then developed using 1:2 cyclohexane:ethyl acetate. Plates were imaged using BioRad Imager and spots were quantified using ImageJ.

### OCR assays

Hep3B cells were cultured on Seahorse 96-well plates at a density of 50,000 cells per well, respectively, in MEM medium supplemented with 10% FBS. On the day prior to analysis, culture medium was replaced with substrate-limited DMEM medium supplemented with 0.5 mM glucose, 1% FBS, 0.5 mM L-carnitine, 1 mM glutamax, pH 7.4 and incubated at 37 °C in a 5% CO_2_ incubator for 24 h. On the day of the assay, media was swapped to fatty acid oxidation buffer (KHB buffer supplemented with 2.5 mM glucose, 0.5 mM L-carnitine, and 5 mM Hepes, pH 7.4), and the cells were placed in a non-CO_2_ incubator for 1 h at 37 °C. Immediately after the incubation, and prior to acquisition, cells were treated with 167 μM of either bovine serum albumin (BSA), BSA-Palmitate Complex (Cayman Chemical), or BSA-Oleate (Cayman Chemical). The OCR was recorded using an XFe96 Seahorse analyzer instrument. Baseline measurements of OCR and ECAR (not reported) were sampled prior to sequential injection of mitochondrial inhibitors: 1.5 μM oligomycin A (ATP synthase complex V inhibitor; MP Biomedicals), 1 μM FCCP (uncoupling agent that collapses the proton gradient; APExBio Technology), and 0.5 μM of rotenone (complex I inhibitor; APExBio Technology) and 0.5 μM of antimycin A (complex III inhibitor; Enzo Life Sciences) to shut down mitochondrial respiration. After the OCR was recorded, each well was fixed, stained with DAPI, and the DAPI intensity was recorded using a Citation 5 microplate reader (Agilent-BioTek). Each well was normalized to the maximum DAPI staining on the plate, and the resulting OCR traces were scaled according to this normalization factor. The Mito FA OCR was determined by calculating the difference of the mitochondria-specific OCR (the difference in nonmitochondrial OCR following rotenone and antimycin A treatment from the uncoupled OCR following FCCP treatment) between cells treated with etomoxir to those treated with DMSO. Four experimental replicates were performed for these assays.

### Lipid droplet isolation

Hep3B cells were grown to confluence in 15 cm dishes and treated with 200 μM OA for 16 h to promote the production of LDs. The cells were then washed twice with ice-cold PBS and harvested using a cell scraper. Cells were pelleted at 250*g* for 10 min at 4 °C and resuspended in five pellet volumes of ice-cold lysis buffer consisting of 20 mM Tris, pH 7.4, 1 mM EDTA. The cell suspension was incubated on ice for 10 min and then lysed by pipetting through a 27-gauge needle five times. Cell lysate was centrifuged for 10 min at 1000*g* at 4 °C to remove nuclei and insoluble cellular aggregates. The supernatant was collected and mixed with ice-cold lysis buffer with 20% sucrose. Cell lysates were then added to an ultracentrifuge tube and 5 ml ice-cold lysis buffer with 5% sucrose was layered gently over the sample. A layer of ice-cold lysis buffer was added over the sucrose layers to fill the tube. The solution was centrifuged for 1 h at 28,000*g* at 4 °C. The floating LD layer was collected and placed in a microfuge tube, which was then centrifuged for 10 min at 20,000*g* at 4 °C. The bottom soluble fraction was removed, and the process was repeated until the LD layer was reduced to a final volume of 50 μl.

### Western blotting

Cells for Western blotting were treated the same way as those for imaging, except, in this case, 200,000 cells were seeded onto a 6-cm plate and the transfection mixtures were doubled in volume. Cells were harvested 3 days post seeding in lysis buffer consisting of TBS supplemented with 1% Triton-X, 2 mM EDTA with 1 mM PMSF. The lysis mixture was pipette mixed and incubated on ice for 30 min. The lysate was then clarified at 12,000*g* for 10 min and the supernatant was removed and mixed with 3× SDS sample buffer. Samples were run on 12% polyacrylamide gels and transferred onto Immobilon-P polyvinylidene difluoride membranes (MilliporeSigma) in 1× transfer buffer with 10% ethanol and 0.01% SDS at 130 mA for 3 h at 4 °C. Membranes were allowed to dry and then blocked for 1 h with 5% BSA in TBS supplemented with 0.1% Tween, before incubating with the specified primary antibodies for 4 h to overnight at 4 °C. Blots were then washed with TBS supplemented with 0.1% Tween and incubated with horseradish peroxidase-linked anti-mouse and anti-rabbit immunoglobulin G secondary antibodies for 30 min at room temperature. Washed blots were developed using the clarity western ECL substrate (Bio-Rad Laboratories) and imaged using a Gel-Doc Imager (Bio-Rad). The relative abundance of proteins was determined by densitometry analysis using the program Fiji.

### Protein expression and purification

#### FL hsDFCP1

Human DFCP1 (Uniprot ID: Q9HBF4) was cloned into a custom vector, where the DNA sequence encoding GFP in a pEGFP-C1 vector was replaced with a DNA sequence encoding a FLAG-TEV sequence using the NheI and BglII restriction sites. For expression of FLAG-DFCP1, 1.25 × 10^6^ Expi293 cells ml^−1^ (Thermo Fisher Scientific), cultured in Expi293 media (Thermo Fisher Scientific) without antibiotic and antimyotics, were transiently transfected with a solution of 1 μg ml^−1^ plasmid DNA and 1 mg ml^−1^ PEI (Polysciences). After 48 h expression, cells were harvested, pelleted, and resuspended in lysis buffer comprised of 25 mM Tris, pH 8.0, 300 mM NaCl, 5% glycerol, 1% Triton-X, 1 mM PMSF, 1× protease inhibitor tablet without EDTA (Thermo Fisher Scientific). Cells were incubated in lysis buffer on ice for 30 min, and insoluble cellular components were removed by centrifugation at 12,000*g* for 10 min. Clarified lysates were incubated with 1 ml Anti-DYKDDDDK (FLAG) Affinity Resin (Genscript) for 2 h at 4 °C and then washed with 15 column volumes (CVs) of wash buffer, comprised of 25 mM Tris, pH 8.0, 300 mM NaCl, 5% glycerol, and 1 mM PMSF, followed by 15 CVs of wash buffer supplemented with 1 mM ATP. Bound DFCP1 was eluted by four sequential 1 h incubations of the FLAG resin with 1 CV of wash buffer supplemented with 1 mg ml^−1^ 3X-FLAG peptide. The elution fractions were pooled, and the excess FLAG peptide was removed by dialysis using a 3-14 kDa cutoff, with a dialysis buffer composed of 25 mM Tris, pH 8, 150 mM NaCl, 1 mM DTT, 1 mM MgCl_2_. The dialyzed protein was spin concentrated using a 30 kDa MWCO ultracentrifugation filter (MilliporeSigma) and subjected to size-exclusion chromatography using a Superose 6 attached to an AKTA Pure 25L (Cytiva Life Sciences) equilibrated in a buffer consisting of 25 mM Tris pH 8, 150 mM NaCl, 1 mM DTT, and 1 mM MgCl_2_. The resulting monomer and dimer peaks were isolated together, spin concentrated using a 30-kDa MWCO ultracentrifugation filter, and flash frozen in liquid nitrogen.

#### DFCP1 NTPase domain and NTPase domain mutations

Residues 112-415 of mouse DFCP1 (Uniprot ID: Q810J8), which contains an NTPase domain that is 98.8% identical to human DFCP1, was inserted into the pMal-c2e bacterial expression vector using the EcoRI and Sal1 restriction enzyme cleavage sites. DFCP1 NTPase mutations K193A and R266Q were introduced using the QuikChange mutagenesis kit (Agilent). These constructs were transformed into *Rosetta* (DE3) cells (Novagen) that were subsequently cultured in Terrific Broth medium that was supplemented with 100 μg ml^−1^ carbenicillin and 100 μg ml^−1^ chloramphenicol at 37 °C. Protein expression was induced with 1 mM IPTG at 18 °C for 16 h. Cells were homogenized in lysis buffer, comprised of 25 mM Tris pH 8.0, 300 mM NaCl, 1 mM MgCl_2_, 4 mM benzamidine hydrochloride, and 1 mM PMSF, and lysed using a microfluidizer 110L (Microfluidics). Lysates were clarified by centrifugation at 16,000*g* for 45 min and the supernatant was loaded onto amylose affinity resin (New England BioLabs). The column was washed with 10 CVs of lysis buffer and the protein was eluted with sequentially added 1 CV aliquots of lysis buffer supplemented with 20 mM maltose. Aliquots containing the eluted protein were combined and passed over a HiLoad 26/600 Superdex 200 pg size-exclusion column (Cytiva) equilibrated in 25 mM Tris pH 8.0, 150 mM NaCl, 1 mM MgCl_2_, and 1 mM DTT. The monomer and dimer peaks were isolated and the protein was spin concentrated to 2 ml volume using a 10 MWCO ultracentrifugation filter. The MBP tag was removed by treating the concentrated protein solution with 0.15 mg ml^−1^ TEV protease overnight at 4 °C. The TEV-cleaved protein was filtered and flashed diluted with two volumes of dialysis buffer lacking NaCl in order to reduce the NaCl concentration to 50 mM. The cleaved protein was further purified with a MonoQ 4.6/100 PE column (Cytiva), using a 50 mM to 300 mM NaCl gradient spanning 25 CVs. The peak corresponding to DFCP1 was isolated, spin concentrated, and then flash frozen in liquid nitrogen.

#### Cdc42

WT human cdc42 (Uniprot ID: P60953) was inserted into the pTYB11 IMPACT bacterial expression vector (New England BioLabs), using the Sal1 and SapI restriction enzyme cleave sites. Cdc42 was transformed into *Rosetta* (DE3) cells (Novagen) that were subsequently cultured in Terrific Broth medium, supplemented with 100 μg ml^−1^ carbenicillin at 37 °C. Protein expression was induced with 1 mM IPTG at 18 °C for 16 h. Cells were homogenized in lysis buffer (25 mM Tris pH 8.0, 300 mM NaCl, 5 mM MgCl_2_, 0.1 mM GDP, 4 mM benzamidine hydrochloride, and 1 mM PMSF) and lysed using a microfluidizer 110L (Microfluidics). Lysates were clarified by centrifugation at 16,000*g* for 45 min, and the supernatant was loaded onto the chitin affinity resin (New England BioLabs). The affinity tag was removed by inducing self-cleavage of the intein domain with 50 mM DTT for 48 h at 4 °C. Proteins were eluted from the column in 25 mM Tris pH 8.0, 300 mM NaCl, 5 mM MgCl_2_, 0.1 mM GDP, and 50 mM DTT. A final size-exclusion chromatography purification step was performed on a HiLoad 26/600 Superdex 75 pg column (Cytiva) equilibrated in 25 mM Tris pH 8.0, 50 mM NaCl, 2 mM MgCl_2_, 0.1 mM GDP, and 1 mM DTT. The protein was spin concentrated using an ultracentrifugation filter and flash frozen in liquid nitrogen.

### Data presentation and statistical methods

In all relevant figure panels, data points from the number of experiments indicated in each figure legend is plotted along with either the mean and SD or the box-and-whisker representation of the data. In all cases of the box-and-whisker representation, the line between the boxes represents the median, the lower and upper boxes represent the first quartile and third quartiles, respectively, and the whiskers represent 1.5 × the inner quartile range (the separation between the upper and lower quartiles). Depending on the nature of the experiment, statistical analyses of the measurements were performed using either the Mann-Whitney U-test, the Wilcoxon matched-pairs signed rank test, or the student’s *t* test (as indicated in each figure legend).

## Data availability

Detailed data are available by request.

## Supporting information

This article contains supporting information.

## Conflict of interest

The authors declare that they have no conflicts of interest with the contents of this article.

## References

[1] Krahmer N., Farese R.V., Walther T.C. (2013). Balancing the fat: lipid droplets and human disease. EMBO Mol. Med..

[2] Schulze R.J., Sathyanarayan A., Mashek D.G. (2017). Breaking fat: the regulation and mechanisms of lipophagy. Biochim. Biophys. Acta Mol. Cell Biol. Lipids.

[3] Singh R., Cuervo A.M. (2012). Lipophagy: connecting autophagy and lipid metabolism. Int. J. Cell Biol..

[4] Kaushik S., Cuervo A.M. (2015). Degradation of lipid droplet-associated proteins by chaperone-mediated autophagy facilitates lipolysis. Nat. Cell Biol..

[5] Schott M.B., Weller S.G., Schulze R.J., Krueger E.W., Drizyte-Miller K., Casey C.A. (2019). Lipid droplet size directs lipolysis and lipophagy catabolism in hepatocytes. J. Cell Biol..

[6] Axe E.L., Walker S.A., Manifava M., Chandra P., Roderick H.L., Habermann A. (2008). Autophagosome formation from membrane compartments enriched in phosphatidylinositol 3-phosphate and dynamically connected to the endoplasmic reticulum. J. Cell Biol..

[7] Ridley S.H., Ktistakis N., Davidson K., Anderson K.E., Manifava M., Ellson C.D. (2001). FENS-1 and DFCP1 are FYVE domain-containing proteins with distinct functions in the endosomal and Golgi compartments. J. Cell Sci..

[8] Bersuker K., Peterson C.W.H., To M., Sahl S.J., Savikhin V., Grossman E.A. (2018). A proximity labeling strategy provides insights into the composition and dynamics of lipid droplet proteomes. Dev. Cell.

[9] Li D., Zhao Y.G., Li D., Zhao H., Huang J., Miao G. (2019). The ER-localized protein DFCP1 modulates ER-lipid droplet contact formation. Cell Rep..

[10] Xu D., Li Y., Wu L., Li Y., Zhao D., Yu J. (2018). Rab18 promotes lipid droplet (LD) growth by tethering the ER to LDs through SNARE and NRZ interactions. J. Cell Biol..

[11] Kiss R.S., Chicoine J., Khalil Y., Sladek R., Chen H., Pisaturo A. (2019). Comparative proximity biotinylation implicates RAB18 in cholesterol mobilization and biosynthesis. bioRxiv.

[12] Hirose H., Arasaki K., Dohmae N., Takio K., Hatsuzawa K., Nagahama M. (2004). Implication of ZW10 in membrane trafficking between the endoplasmic reticulum and Golgi. EMBO J..

[13] Gao G., Sheng Y., Yang H., Chua B.T., Xu L. (2019). DFCP1 associates with lipid droplets. Cell Biol. Int..

[14] Cui W., Sathyanarayan A., Lopresti M., Aghajan M., Chen C., Mashek D.G. (2021). Lipophagy-derived fatty acids undergo extracellular efflux via lysosomal exocytosis. Autophagy.

[15] Li Z., Weller S.G., Drizyte-Miller K., Chen J., Krueger E.W., Mehall B. (2020). Maturation of lipophagic organelles in hepatocytes is dependent upon a Rab10/dynamin-2 complex. Hepatology.

[16] Niso-Santano M., Malik S.A., Pietrocola F., Bravo-San Pedro J.M., Mariño G., Cianfanelli V. (2015). Unsaturated fatty acids induce non-canonical autophagy. EMBO J..

[17] Nguyen T.B., Louie S.M., Daniele J.R., Tran Q., Dillin A., Zoncu R. (2017). DGAT1-Dependent lipid droplet biogenesis protects mitochondrial function during starvation-induced autophagy. Dev. Cell.

[18] Cheung P.C., Trinkle-Mulcahy L., Cohen P., Lucocq J.M. (2001). Characterization of a novel phosphatidylinositol 3-phosphate-binding protein containing two FYVE fingers in tandem that is targeted to the Golgi. Biochem. J..

[19] Bourne H.R., Sanders D.A., McCormick F. (1991). The GTPase superfamily: conserved structure and molecular mechanism. Nature.

[20] Mohanakrishnan A., Tran T.V.M., Kumar M., Chen H., Posner B.A., Schmid S.L. (2017). A highly-sensitive high throughput assay for dynamin’s basal GTPase activity. PLoS One.

[21] Sigal I.S., Gibbs J.B., D’Alonzo J.S., Scolnick E.M. (1986). Identification of effector residues and a neutralizing epitope of Ha-ras-encoded p21. Proc. Natl. Acad. Sci. U. S. A..

[22] Babst M. (1998). The Vps4p AAA ATPase regulates membrane association of a Vps protein complex required for normal endosome function. EMBO J..

[23] Damke H., Baba T., Warnock D.E., Schmid S.L. (1994). Induction of mutant dynamin specifically blocks endocytic coated vesicle formation. J. Cell Biol..

[24] Matveeva E.A., He P., Whiteheart S.W. (1997). N-ethylmaleimide-sensitive fusion protein contains high and low affinity ATP-binding sites that are functionally distinct. J. Biol. Chem..

[25] Moss T.J., Andreazza C., Verma A., Daga A., McNew J.A. (2011). Membrane fusion by the GTPase atlastin requires a conserved C-terminal cytoplasmic tail and dimerization through the middle domain. Proc. Natl. Acad. Sci. U. S. A..

[26] Ford M.G.J., Jenni S., Nunnari J. (2011). The crystal structure of dynamin. Nature.

[27] Cotte A.K., Aires V., Fredon M., Limagne E., Derangère V., Thibaudin M. (2018). Lysophosphatidylcholine acyltransferase 2-mediated lipid droplet production supports colorectal cancer chemoresistance. Nat. Commun..

[28] Pfisterer S.G., Gateva G., Horvath P., Pirhonen J., Salo V.T., Karhinen L. (2017). Role for formin-like 1-dependent acto-myosin assembly in lipid droplet dynamics and lipid storage. Nat. Commun..

[29] Filadi R., Greotti E., Turacchio G., Luini A., Pozzan T., Pizzo P. (2015). Mitofusin 2 ablation increases endoplasmic reticulum–mitochondria coupling. Proc. Natl. Acad. Sci. U. S. A..

[30] Niu L., Ma T., Yang F., Yan B., Tang X., Yin H. (2019). Atlastin-mediated membrane tethering is critical for cargo mobility and exit from the endoplasmic reticulum. Proc. Natl. Acad. Sci. U. S. A..

[31] Limoges M.-A., Cloutier M., Nandi M., Ilangumaran S., Ramanathan S. (2021). The GIMAP family proteins: an incomplete puzzle. Front. Immunol..

[32] Schwefel D., Arasu B.S., Marino S.F., Lamprecht B., Köchert K., Rosenbaum E. (2013). Structural insights into the mechanism of GTPase activation in the GIMAP family. Structure.

[33] Schwefel D., Frohlich C., Eichhorst J., Wiesner B., Behlke J., Aravind L. (2010). Structural basis of oligomerization in septin-like GTPase of immunity-associated protein 2 (GIMAP2). Proc. Natl. Acad. Sci. U. S. A..

[34] He S., Ni D., Ma B., Lee J.-H., Zhang T., Ghozalli I. (2013). PtdIns(3)P-bound UVRAG coordinates Golgi–ER retrograde and Atg9 transport by differential interactions with the ER tether and the beclin 1 complex. Nat. Cell Biol..

