# Supplementary material for: The NTPase activity of the double FYVE domain–containing protein 1 regulates lipid droplet metabolism
Source: J Biol Chem. 2022 Dec 24;299(2):102830. doi: 10.1016/j.jbc.2022.102830 (PMC9881219; doi:10.1016/j.jbc.2022.102830)
Supplement: Supplemental Figure Legends [file mmc1.docx]

**Supplemental Information**

**Title:** The NTPase Activity of the Double FYVE Domain Containing Protein 1 (DFCP1) Regulates Lipid Droplet Metabolism.

Ismail, V.A., Naismith, T. and Kast, D.J.

Dept. of Cell Biology and Physiology, Washington University School of Medicine, St. Louis MO

**Supplemental Figure Legends:**

**Figure S1: DFCP1 Localizes to LDs and Autophagosomes.**

**(A)** Confocal images of fixed U2OS cells stained with DAPI, LipidTOX Deep Red and anti-DFCP1 antibody (with an Alexa Fluor 488 secondary antibody). Prior to fixation, cells were either treated with ethanol (left) or OA (middle and right) for 4 h before incubating in either growth media for 1 h (left and middle), or growth media supplemented with 1 μM Wortmannin for 30 min (right).

**(B)** Average of intensity line scans across LDs (*n*=15) from **A** (middle).

**(C)** Control cells for the endogenous staining of DFCP1. (Left) Fixed U2OS cells expressing FLAG-DFCP1 and stained with DAPI, anti-FLAG (conjugated to an Alexa Fluor 555 secondary antibody), and anti-DFCP1 (conjugated to an Alexa Fluor 488 secondary antibody), shows that the DFCP1 antibody recognizes the exogenous FLAG-DFCP1. (Right) By contrast, the cytoplasmic DFCP1 staining is ablated in fixed DFCP1 KD U2OS cells stained with phalloidin and anti-DFCP1.

(**D**) Western blots of clarified lysates harvested from Hep3B cells transiently expressing GFP and GFP-DFCP1, showing that GFP-DFCP1 is 4.1-fold higher than endogenous DFCP1.

**(E)** Western blots of purified LDs isolated from fed and starved OA-stimulated U2OS cells expressing GFP-DFCP1 showing the total cell lysate (L), the last wash (W), and the purified LD fraction (LD). Note, the LD fraction lacks both the cytosolic (GAPDH) and ER (calreticulin) markers, but still retains GFP-DFCP1.

**(F)** Confocal images of purified LDs from **E** and stained with LipidTOX Deep Red (left). The scale bars represent 10 μm for the full field-of-view and 2 μm for the inset. The accumulation of GFP-DFCP1 on each LD, as determined by the log of maximum fluorescence intensity of GFP-DFCP1 from 10 equally and radially spaced line scans through that LD, is plotted along with a box-and-whisker representation of the data on the right.

**(G)** Western blot showing the conversion of endogenous LC3-I to LC3-II in clarified cell lysates from NT and KD U2OS cells that were either fed or starved for 4 h prior to harvesting. Graph shows individual measurement of the LC3I/II ratio (determined by densitometry) along with the mean ± SD.

(**H**) Live cell confocal images of U2OS cells expressing LifeAct-BFP and GFP-LC3 and stained with LipidTOX Deep Red. Cells were treated with 200 μM oleic acid (OA) for 4 h prior to 1 h incubations in growth (left) or starvation media (middle). The Pearson’s correlation coefficient of LC3 with LDs measured for each fed and starved U2OS cell is plotted along with a box-and-whisker representation of the data on the right.

The scale bars in whole-cell and inset images represent 10 and 2 μm, respectively.

The statistical significance of the measurements was determined using the Mann–Whitney U-test (**F, H**) or Wilcoxon matched-pairs signed rank test (**G**), based on the indicated number of observations (indicated in the figure panel) recorded from at least two independent transfections. Exact *p*-values are reported with exception to *p*>0.05, which are considered to be nonsignificant (n.s.).

**Figure S2: DFCP1 Regulates LD Metabolism.**

**(A)** Number and diameter distributions of LDs quantified from live-cell confocal images of fed and starved untransfected OA-stimulated Hep3B cells stained with LipidTOX Deep Red. The number of LDs per cell (left) and the individual LD diameters (right) are plotted along with the box-and-whisker representations of the data.

(**B**) Seahorse assays showing the cellular oxygen consumption rate (OCR) for control (black) and DFCP1 KD Hep3B cells (magenta) stimulated with BSA (left) or BSA-oleate complex (middle), and in the presence of DMSO (solid line) or etomoxir (ETO; dashed line). The OCR traces are presented as mean ± SD. The Mito FA OCR, calculated from the difference in the average mitochondria-specific uncoupled OCR (following FCCP) between DMSO and Etomoxir treated cells, is shown as individual measurements along with mean ± SD on the right.

(**C**) Seahorse assays showing the cellular OCR for DFCP1 KD Hep3B cells rescued with GFP (gray) GFP-DFCP1 (teal) stimulated with palmitic acid (PA) and in the presence of DMSO (solid line) or Etomoxir (ETO; dashed line). The OCR traces are presented as mean ± SD. Quantification of the Mito FA OCR is shown as individual measurements along with mean ± SD on the right.

The statistical significance of the measurements in **A** was determined using the Mann–Whitney U-test on the indicated number of observations from at least two independent transfections. The statistical significance in **B** and **C** was determined using a Student’s t-test on 3 independent experiments recorded on the same assay plate. Exact *p*-values are reported with exception to *p*>0.05, which is considered to be nonsignificant (n.s.).

**Figure S3: Domain Requirements for DFCP1 Localization.**

**(A)** Domain diagram of DFCP1 depicting the GFP-tagged constructs used in this figure.

**(B)** Western blot showing expression levels of the all GFP-DFCP1 truncations depicted in **A** in U2OS cells.

**(C-G)** Representative images of U2OS cells expressing LifeAct-mTagBFP2 and either GFP-Sec61β (**C**) or the indicated GFP-DFCP1 truncations (**D**-**G**). Prior to imaging, all cells were treated with OA for 20 h before incubating in either growth (fed) or starvation media (starved) for 18 h. LDs were stained by treating cells with LipidTOX Deep Red for 30 min. The scale bars in whole-cell and inset images represent 10 and 2 μm, respectively.

**(H)** The extent of colocalization (Pearson’s correlation coefficient) between GFP-DFCP1 and LDs from cell populations depicted in **Figures 3C-F** and **Figures S3C-G.** The extent of colocalization between GFP-Sec61B and LDs is also included as a reference. Individual colocalization measurements for each cell are plotted along with a box-and-whisker representation of the data. The statistical significance was determined using the Mann–Whitney U-test for the indicated number of observations from 2 independent transfections. The exact *p*-values are reported.

**Figure S4: Characterization of the DFCP1 NTPase Domain.**

**(A)** The percent identity across species for the full length human DFCP1 protein (open bars) and the DFCP1 NTPase domain (striped bars), showing that the NTPase domain is highly conserved.

**(B)** The average number of residues separating N-box 1 and N-box 4 in the indicated family of NTPases. Notably, the DFCP1 NTPase domain has a separation similar to that of the Ras superfamily GTPases.

**(C)** Comparison of the specific GDP release rates for 10 μM Cdc42 and the constructs in **Figure 4C** and **4D** after 4 h incubations. In the case of WT+E and WT+P_i_, 30mM EDTA or 50mM phosphate buffer, pH 7.4 was included in the assay, respectively. Bar graphs represent mean ±SD for 3 independent experiments.

**(D)** Chart of the frequency of somatic cancer mutations in DFCP1 found tumor samples curated by TCGA (<https://www.cancer.gov/tcga>).

**Figure S5: The NTPase Activity of DFCP1 Modulates LD Accumulation.**

**(A)** Western blots of clarified cell lysates from the rescued DFCP1 KD Hep3B cells depicted in **Figure 5A-D**, showing the relative expression levels of the GFP-DFCP1 mutants after starvation.

(**B**) Seahorse assays showing the cellular oxygen consumption rate (OCR) for Hep3B cells expressing GFP (gray), GFP-DFCP1 WT (black), GFP-DFCP1 K193A (blue), and GFP-DFCP1 R266Q (orange) stimulated with BSA-palmitate complex and in the presence of DMSO (solid line) or etomoxir (ETO; dashed line). The OCR traces are presented as mean ± SD. Quantification of the Mito FA OCR is shown as individual measurements along with mean ± SD on the right. The statistical significance was determined using a Student’s t-test on 3 independent experiments recorded on the same assay plate. Exact *p*-values are reported.
