# Supplementary figures and images for: The NTPase activity of the double FYVE domain–containing protein 1 regulates lipid droplet metabolism
Source: J Biol Chem. 2022 Dec 24;299(2):102830. doi: 10.1016/j.jbc.2022.102830 (PMC9881219; doi:10.1016/j.jbc.2022.102830)

**Figure S1**

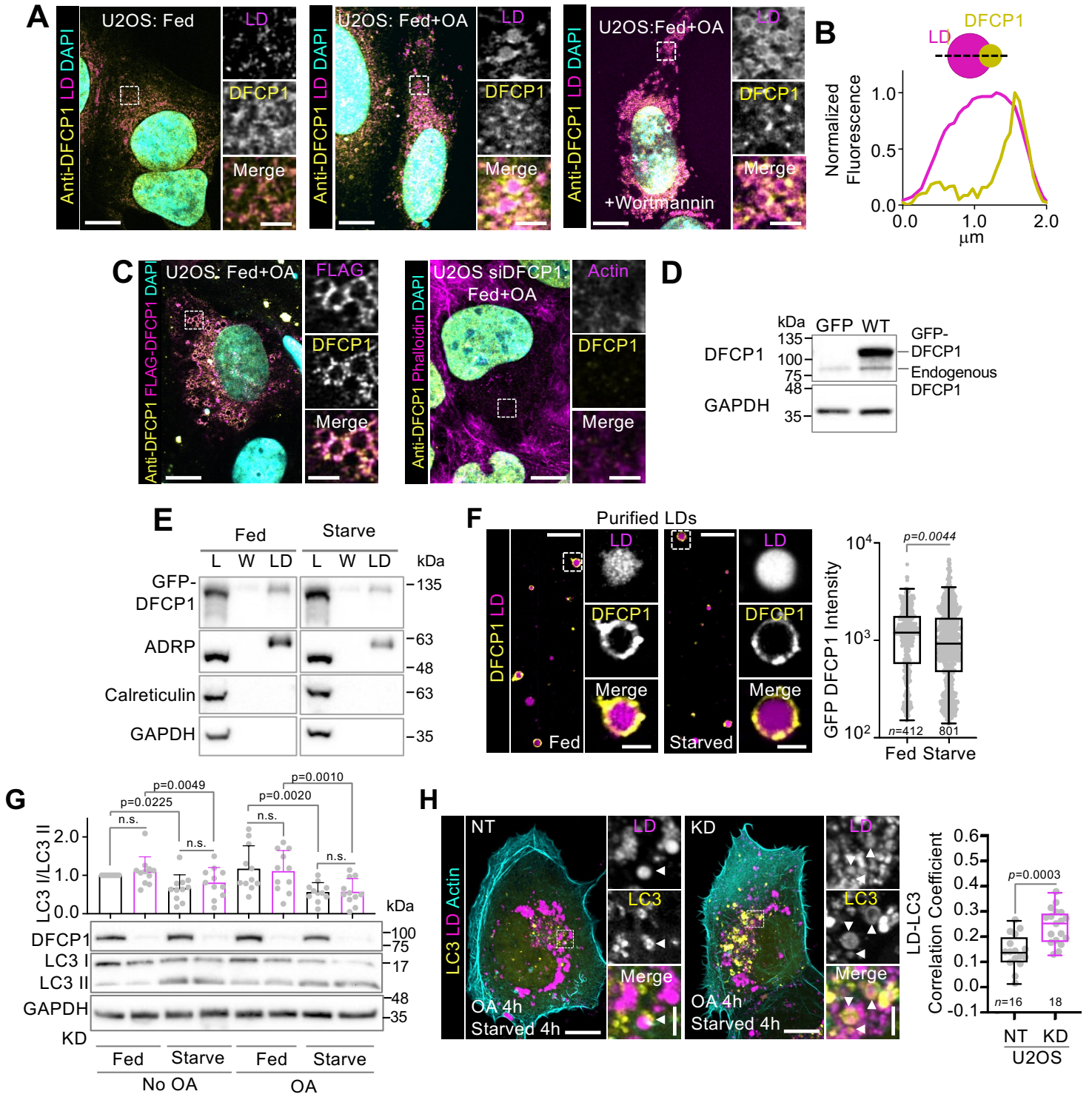

**Figure S2**

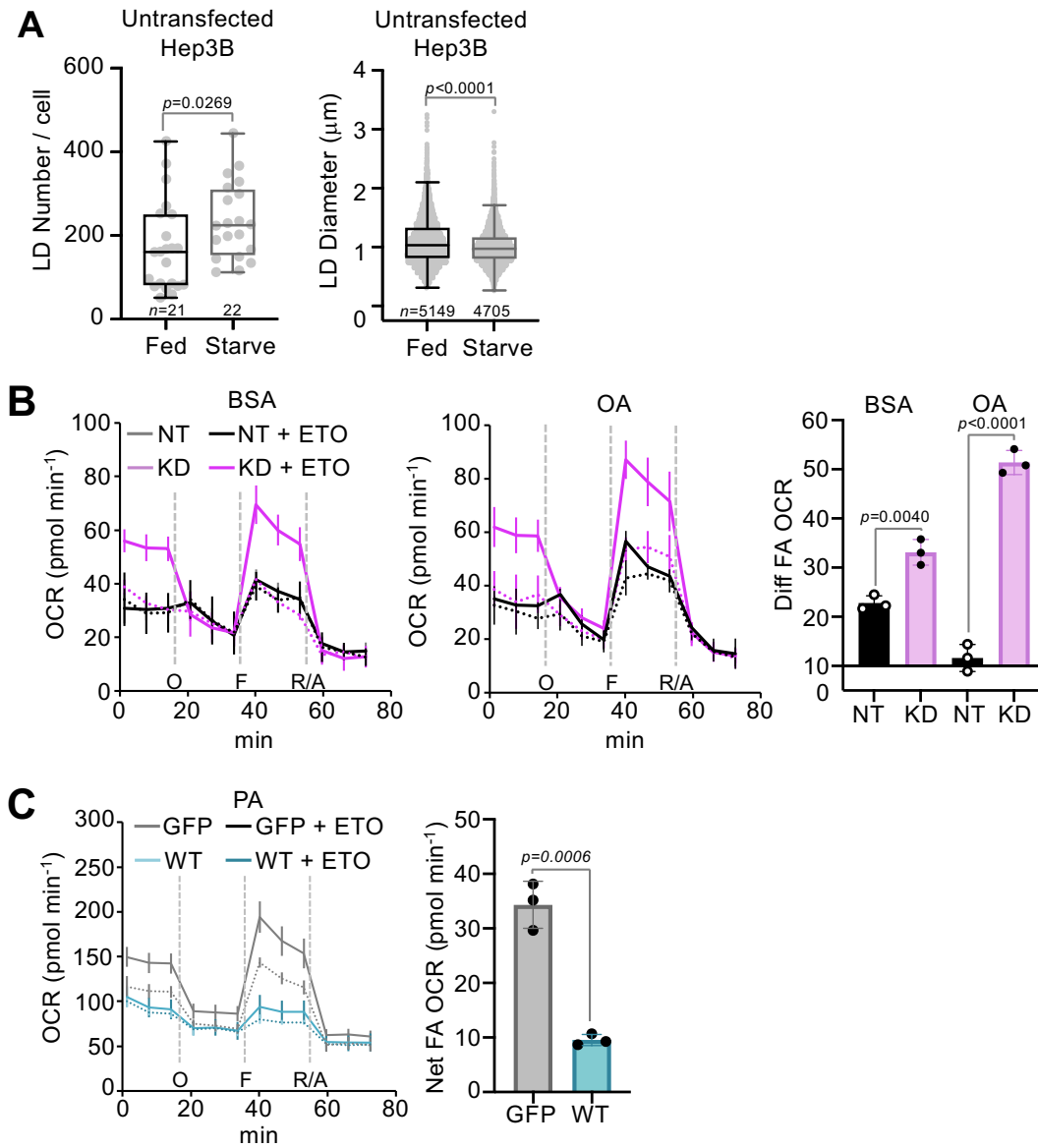

**Figure S3**

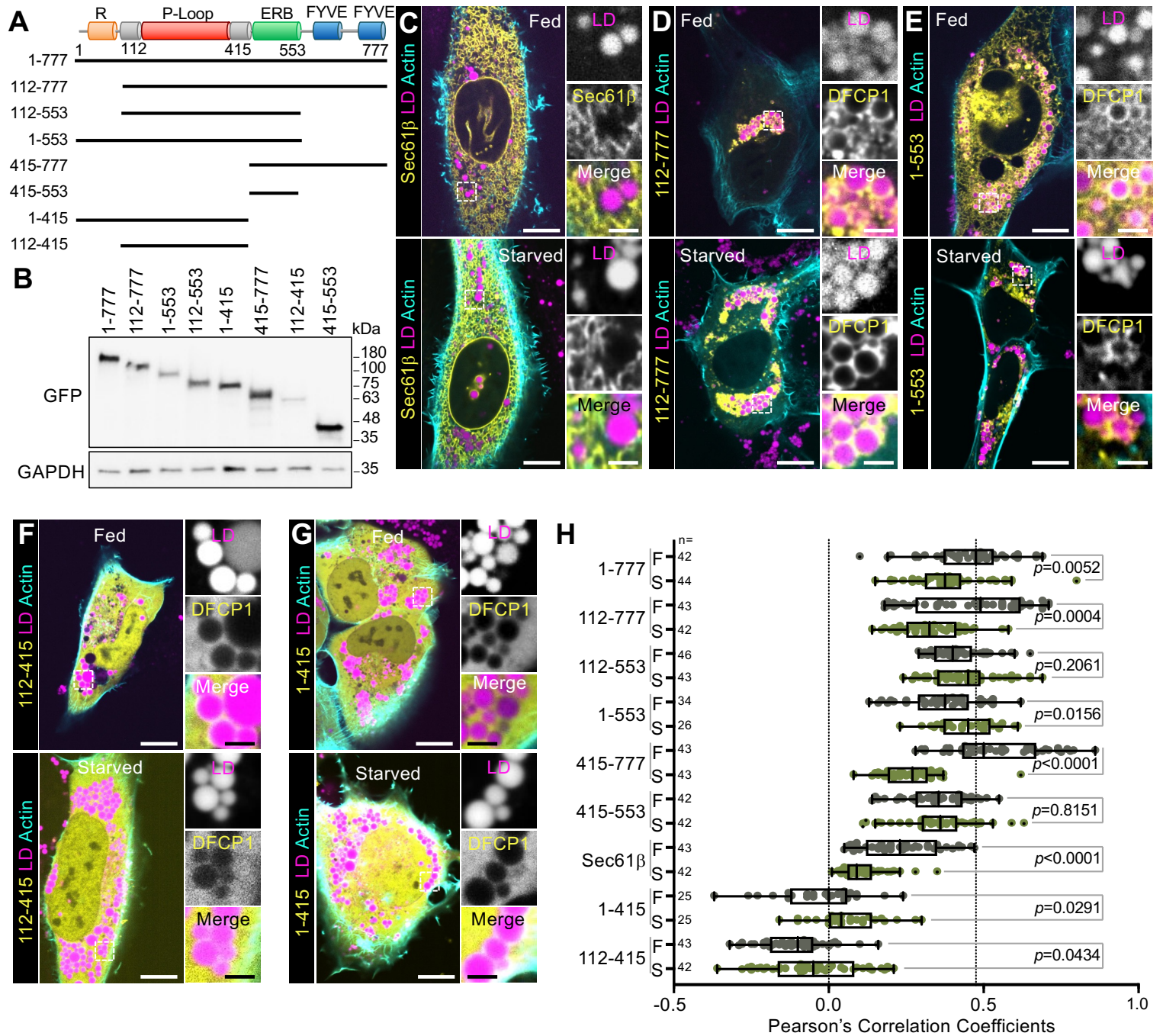

Figure S4

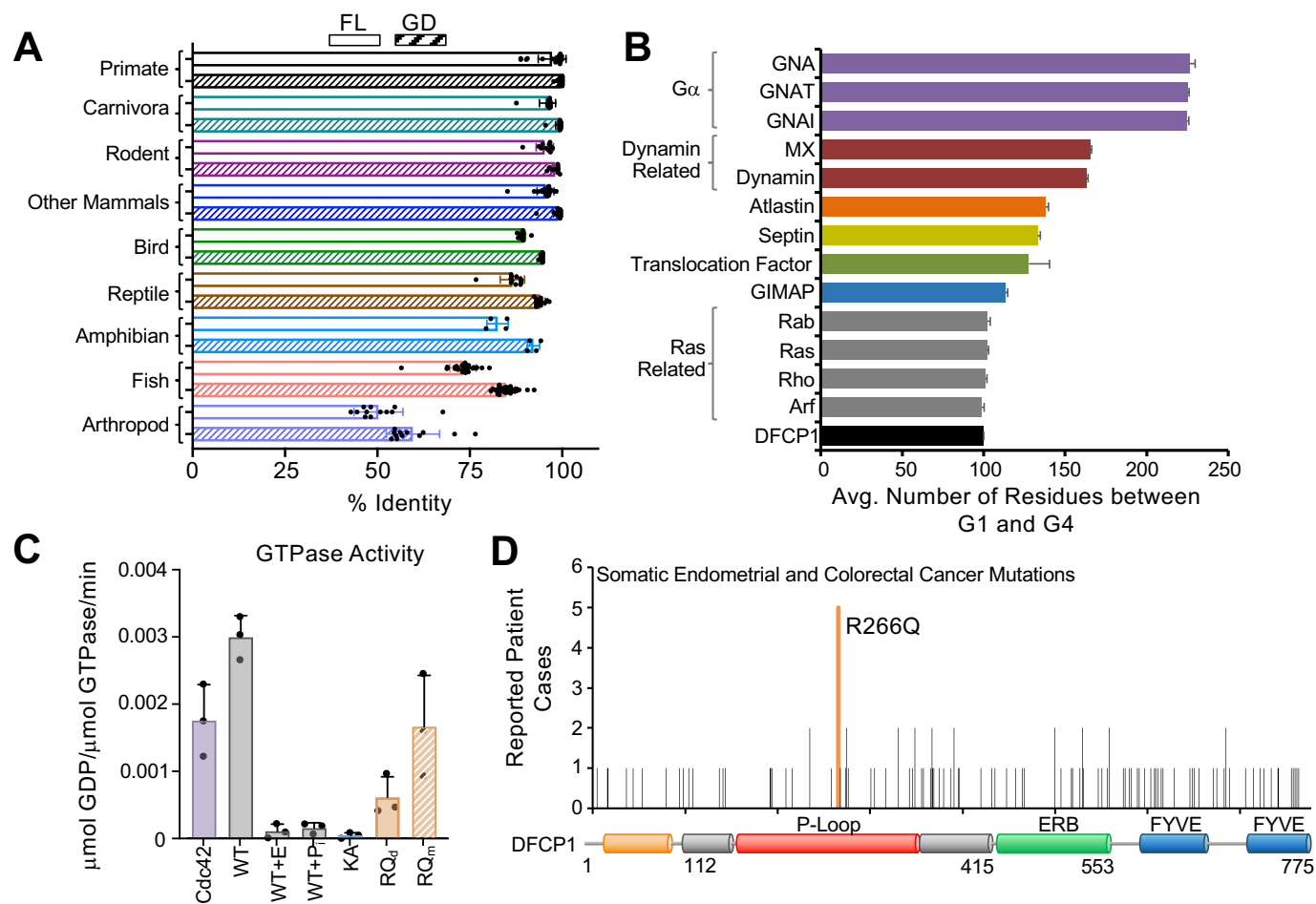

**Figure S5**

**A**

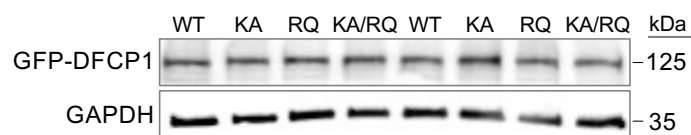

**B**

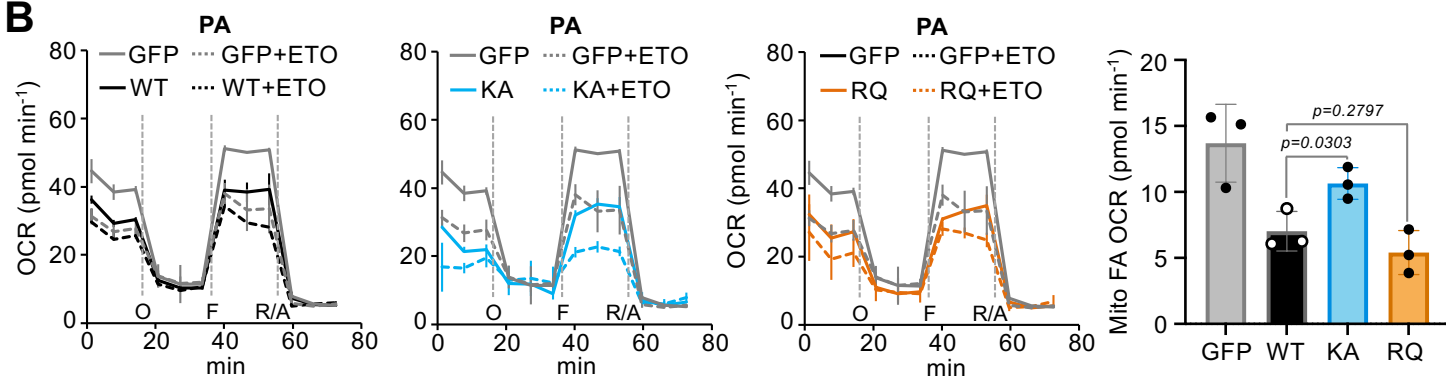

Supplement: Supplemental Figures S1–S5 [file mmc2.pdf]
